# Mutational Mtc6p attenuates autophagy and improves secretory expression of heterologous proteins in *Kluyveromyces marxianus*

**DOI:** 10.1186/s12934-018-0993-9

**Published:** 2018-09-14

**Authors:** Yang Liu, Wen-Juan Mo, Tian-Fang Shi, Meng-Zhu Wang, Jun-Gang Zhou, Yao Yu, Wen-Shan Yew, Hong Lu

**Affiliations:** 1 State Key Laboratory of Genetic Engineering, School of Life Science, Fudan University, Shanghai, 200438 People’s Republic of China; 2 Shanghai Engineering Research Center of Industrial Microorganisms, Shanghai, 200438 People’s Republic of China; 3 NUS Synthetic Biology for Clinical and Technological Innovation, 28 Medical Drive, Singapore, 117456 Singapore; 4 Department of Biochemistry, Yong Loo Lin School of Medicine, National University of Singapore, 8 Medical Drive, Singapore, 117597 Singapore

**Keywords:** *Kluyveromyces marxianus*, Heterologous protein expression, Autophagy, *MTC6*, Ruminal feruloyl esterase

## Abstract

**Background:**

The yeast *Kluyveromyces marxianus* is an emerging cell factory for heterologous protein biosynthesis and its use holds tremendous advantages for multiple applications. However, which genes influence the productivity of desired proteins in *K. marxianus* has so far been investigated by very few studies.

**Results:**

In this study, we constructed a *K. marxianus* recombinant (FIM1/Est1E), which expressed the heterologous ruminal feruloyl esterase Est1E as reporter. UV-^60^Co-γ irradiation mutagenesis was performed on this recombinant, and one mutant (be termed as T1) was screened and reported, in which the productivity of heterologous Est1E was increased by at least tenfold compared to the parental FIM1/Est1E recombinant. Transcriptional perturbance was profiled and presented that the intracellular vesicle trafficking was enhanced while autophagy be weakened in the T1 mutant. Moreover, whole-genome sequencing combined with CRISPR/Cas9 mediated gene-editing identified a novel functional protein Mtc6p, which was prematurely terminated at Tyr251 by deletion of a single cytosine at 755 loci of its ORF in the T1 mutant. We found that deleting C755 of *MTC6* in FIM1 led to 4.86-fold increase in the production of Est1E compared to FIM1, while the autophagy level decreased by 47%; on the contrary, when reinstating C755 of *MTC6* in the T1 mutant, the production of Est1E decreased by 66% compared to T1, while the autophagy level increased by 124%. Additionally, in the recombinant with attenuated autophagy (i.e., FIM1 *mtc6*^C755Δ^ and T1) or interdicted autophagy (i.e., FIM1 *atg1*Δ and T1 *atg1*Δ), the productivity of three other heterologous proteins was also increased, specifically the heterologous mannase Man330, the β-1,4-endoxylanase XynCDBFV or the conventional EGFP.

**Conclusions:**

Our results demonstrated that Mtc6p was involved in regulating autophagy; attenuating or interdicting autophagy would dramatically improve the yields of desired proteins in *K. marxianus*, and this modulation could be achieved by focusing on the premature mutation of Mtc6p target.

**Electronic supplementary material:**

The online version of this article (10.1186/s12934-018-0993-9) contains supplementary material, which is available to authorized users.

## Background

*Kluyveromyces marxianus* is a generally regarded as safe (GRAS) ascomycetous yeast that has advantageous properties for multiple applications. *K. marxianus* has been employed as a host for synthesizing a number of heterologous agro-industrial and pharmaceutical proteins, such as the GOX, the Cu/Zn SOD and the Dengue virus type 1 NS1 [1–3]. However, the maximum reported yields of these proteins produced by *K. marxianus* are still within the range of milligrams per litre and require substantial improvements before sustainable industrial utility can be attained [4–6].

Accumulating evidences indicate that the translational and post-translational efficiency and/or the vesicle trafficking capacity play an important role in the expression of membrane or secretory proteins [7]. However, protein target to secretory pathways often suffer from secretion saturation [8, 9]. Strategies, such as engineering protein folding or modulating vesicular trafficking, have been carried out in *S. cerevisiae* or *P. pastoris* to improve the production of desired proteins [10, 11]. For instance, many chaperones or redox enzymes, such as the chaperone BiP or the protein disulfide isomerase Pdi1p, have been used to assist protein folding; some components, such as the SNAREs elements which are engaged in the ER-to-Golgi trafficking, have been modulated and modified to increase the secretory expression of desired proteins.

Generally, anterograde transport of the correctly folded precursor proteins from ER to *cis*-Golgi are carried by the COPII-coated vesicles, which are mainly organized by Sar1p, Sec16p, Sec23p/Sec24p and Sec13p/Sec31p, and budding at the ER exit sites (ERES) [7]. The ER can also act as the primary site for autophagosome biogenesis, and some interactions between the constituents of ERES and the Atg machinery have been identified [12, 13]. For instance, the Atg1p, an essential kinase in initiating and regulating autophagy, could phosphorylate Sec23Ap and reduce cargo export at the ERES, while it could also phosphorylate Sec16p and modulate the morphology of ERES [14, 15]. Besides, formation of the pre-autophagosomal structures (PAS) requires both COPI and COPII vesicles, in which some components of the COPII vesicles (e.g., Sar1p, Sec24p and Ypt1p/Rab1p) serve as multitasking proteins in the secretion and autophagy cross-talk [16]. However, in the previous studies, the equilibrium and interactions between autophagy and secretion of proteins have been largely neglected in most engineering efforts for improving the yields of desired proteins from these eukaryotic expression systems, and the complex protein secretory pathway in *K. marxianus* remains elusive.

Here, we aimed to improve the yields of desired proteins expressed in *K. marxianus* using the conventional irradiation mutagenesis, and attempted to optimize the strategies for yeast engineering by identifying the major genetic determinants, which play an important role in regulating the productivity of desired proteins in *K. marxianus*.

## Methods

### Strains and cultivations

All the strains and mutants used in this work were described in Table 1. The patent *K. marxianus* FIM1 strain (*CGMCC No. 10621*) was employed, which has been deposited in the China General Microbiological Culture Collection Center (CGMCC). Yeast cells were routinely cultivated in complex YPD medium (10 g/L yeast extract, 20 g/L hipolypepton and 20 g/L glucose), and the recombinants were screened from the synthetic SD-URA medium (10 g/L glucose, 6.7 g/L yeast nitrogen base, 40 mg/L histidine, 40 mg/L leucine and 40 mg/L tryptophan) without uracil due to its intrinsic auxotrophy (*ura3*Δ). For solid media, 2% agar was added. For protein production, the recombinants were cultured in 50 mL of 24-medium (20 g/L yeast extract and 40 g/L glucose) in Erlenmeyer flasks for 96 h at 30 °C, 220 rpm, unless special emphasized. The *E. coli* strain DH5α was utilized for plasmid propagation and maintenance, which were grown in Luria–Bertani medium supplemented with 100 μg/mL of ampicillin when necessary.

**Table 1:** *K. marxianus* strains and mutants used in this study

Names	Genotypes or descriptions	Sources
FIM1	*ura3*Δ	This lab
T1	*ura3*Δ; efficiently secrete and express the desired proteins	This study
FIM1 *cms1*Δ	*ura3*Δ *cms1*Δ	This study
FIM1 *idp1*Δ	*ura3*Δ *idp1*Δ	This study
FIM1 *cda2*Δ	*ura3*Δ *cda2*Δ	This study
FIM1 *hrd3*Δ	*ura3*Δ *hrd3*Δ	This study
FIM1 *ynr021w*Δ	*ura3*Δ *ynr021w*Δ	This study
FIM1 *mtc6*^721−903Δ^	*ura3*Δ *mtc6*^721−903Δ^	This study
FIM1 *mtc6*^C755Δ^	*ura3*Δ *mtc6*^C755Δ^; mimic the frameshift mutation of *MTC6* in T1 mutant	This study
FIM1 *mtc6*^754−1773Δ^	*ura3*Δ *mtc6*^754−1773Δ^; resulting in premature termination at Ser252 of Mtc6p	This study
FIM1 mtc6^S252^*	*ura3*Δ *mtc6*^C755A/T756A^; resulting in premature termination at Ser252 of Mtc6p	This study
FIM1 *mtc6*^G59A^	*ura3*Δ *mtc6*^G59A^; resulting in premature termination at Cys19 of Mtc6p	This study
FIM1 *ssk1*Δ	*ura3*Δ *ssk1*Δ	This study
FIM1 *erf4*Δ	*ura3*Δ *erf4*Δ	This study
FIM1 *sea3*Δ	*ura3*Δ *sea3*Δ	This study
FIM1 *twf1*Δ	*ura3*Δ *twf1*Δ	This study
FIM1 *sea3*Δ *mtc6*^721−903Δ^	*ura3*Δ *sea3*Δ *mtc6*^721−903Δ^	This study
FIM1 *sea3*Δ *mtc6*^721−903Δ^ *ynr021w*Δ	*ura3*Δ *sea3*Δ *mtc6*^721−903Δ^ *ynr021w*Δ	This study
FIM1 *atg1*Δ	*ura3*Δ *atg1*Δ	This study
T1 *atg1*Δ	*ura3*Δ *atg1*Δ; T1 mutant as background	This study
FIM1 *mtc6*^C755Δ^ *atg1*Δ	*ura3*Δ *mtc6*^C755Δ^ *atg1*Δ	This study
T1 *MTC6*	*ura3*Δ *mtc6*^C755Δ^::*MTC6*; T1 mutant as background	This study
FIM1^EA^	*ura3*Δ *ATG8::EGFP*-*ATG8*	This study
FIM1^EA^ *mtc6*^C755Δ^	*ura3*Δ *ATG8::EGFP*-*ATG8 mtc6*^C755Δ^	This study
FIM1^EA^ *atg1*Δ	*ura3*Δ *ATG8::EGFP*-*ATG8 atg1*Δ	This study
T1^EA^	*ura3*Δ *ATG8::EGFP*-*ATG8*; T1 mutant as background	This study
T1^EA^ *mtc6*^C755Δ^	*ura3*Δ *ATG8::EGFP*-*ATG8 mtc6*^C755Δ^; T1 mutant as background	This study
T1^EA^ *atg1*Δ	*ura3*Δ *ATG8::EGFP*-*ATG8 atg1*Δ; T1 mutant as background	This study

### Recombination and gene-editing

For heterologous expression in *K. marxianus*, the heterologous genes (such as *Est1E*, *Man330*, *XynCDBFV* or *EGFP*) were amplified and assembled into the pUKD-N112 plasmid containing *URA3* as biomarker, in which expression of heterologous genes could been driven by the *K. marxianus INU1* promoter, secretory signal peptide and terminator. For genomic engineering (such as deletion, directed mutation or insertion) in *K. marxianus*, the CRISPR/Cas9 plasmids were constructed using the pUKD-N122-AUC plasmid as backbone, which synchronously expressed the Cas9 endonuclease and the sgRNAs. The matched donor sequences could also be designed, amplified and co-transformed with its corresponding CRISPR/Cas9 plasmid. Transformation of the indicated plasmids and/or oligonucleotide fragments into *K. marxianus* was mediated by LiAc/carrier ssDNA/PEG according to the reports with minor modifications [17, 18]. All the primers and DNA sequences were listed in Additional file 1: Table S2.

### Quantification of recombinant proteins

In this work, the yields of desired proteins was measured by the specific enzymatic activity or autofluorescence. For quantifying the extracellular proteins, the samples were harvested by centrifugation from the fermentation supernatant. Activity of the feruloyl esterase Est1E was detected using a spectrophotometer with 2-chloro-4-nitropheyl ferulate (CNPF) as substrate, which would be catalyzed by feruloyl esterase and release chromophores for quantification [19]. Briefly, the crude Est1E was diluted to optimal concentration with 1× PBST (pH6.4, T: 2.5% TritonX-100), and 20 μL of each dilution was transferred into 180 μL of 1 mM CNPF solution while the 2-chloro-4-nitrophenol was regarded as standards, and the specific absorbance under 410 nm were detected using a robotic Microplate-reader following incubation at 37 °C for 10 min. It is worth mentioning that the enzymatic activity unit (U) of Est1E in this work was defined as one nmol of chromophore be released from the substrate per minute under 37 °C, pH 6.4.

Activity of mannase Man330 or β-1,4-endoxylanase XynCDBFV was assayed by measuring the amount of reducing sugars released from enzymatic hydrolysis using the dinitrosalicylic method as reported previously [20, 21]. Briefly, the 0.3% locust bean gum (pH 9.5) was introduced as substrate for mannase, and the 2% xylan (pH 5.5) was used as substrate for β-1,4-endoxylanase. Of note, the enzymatic activity unit (U) of Man330 or XynCDBFV was defined as the amount of enzyme that produced one μmol of reducing sugar per minute under the suitable temperature and pH.

Moreover, EGFP accumulated in the supernatant was quantified by measuring its fluorescence intensity under excitation wavelength 485 nm and emission wavelength 525 nm using a *Tecan Infinite multimode reader*.

### Irradiation mutagenesis and high-throughput screening

The FIM1/Est1E recombinants in logarithmic phase were harvested and exposed to UV radiation at a distance of 20 cm for 30 min using a UV stratalinker (120 mJ/cm^2^), followed by cultivation at 30 °C, 220 rpm for 1 h and stored at 4 °C, overnight. All these manipulations were performed away from light and repeated for a week. Then, the treated cells were inoculated into 50 mL of YPD medium and cultivated to logarithmic phase, and exposed to ^60^Co-γ irradiation at a dose of 14.5 kGy/h for 1 h. The suspension was diluted with ddH_2_O and spread onto SD-URA plates to isolate single colony.

For screening the desired mutants, the heterologous feruloyl esterase Est1E was elected as a biomarker and the artificial CNPF was designed as specific substrate applied to high-throughput screening. Shortly, the single colony was inoculated into 600 μL of 24-medium in 24-well clusters, and incubated at 30 °C, 220 rpm for 96 h. Then, activity of Est1E in the fermentation supernatant was determined as mentioned above.

### Genome and transcriptome analyses

Genomic DNA was extracted using the TIANamp Yeast DNA Kit (*TIANGEN#DP307*) according to the manufacturer’s instructions, and 2 μg of the certified DNA from each sample was submitted for sequencing at the Chinese National Human Genome Center (Shanghai) using the Illumina PET HiSeq technology. The reads were optimized and assembled into eight ungapped contigs with an average coverage of 100×, in turn, a number of SNPs or indels in the mutants against the wild-type strain were identified, then be annotated and assigned by GO and KOG database.

For transcriptome profiling, recombinants were grown in 50 mL of 24-medium in Erlenmeyer flasks and sampled at the indicated time-points (the 4th, 6th, 12th, 24th, 48th and 72th h over the growth). Total RNA was isolated and purified using the ZR Fungal/Bacterial RNA MiniPrep™ (*ZYMO RESEARCH#R2014*) according to the manufacturer’s instructions, and about 10 μg of the validated RNA from each sample was submitted for sequencing at Genergy Biotechnology (Shanghai) Co., Ltd using the Illumina HiSeq 3000. Subsequently, 11.6–38.2 million of read pairs were obtained for each sample and the raw reads were mapped to the FIM1 reference genome, with 79.8–96.9% of the reads successfully mapped. *Cuffnorm* was used to calculate FPKM values, and the differential expression among two samples was analyzed using *DESeq2*, from where the differential expression level of genes (log_2_FoldChange) and corresponding significant levels (adjust *p*-values) were obtained.

### SDS-PAGE and western blot assay

SDS-PAGE and western blot were performed comply with a standard protocol. The cell lysates were prepared using lysis buffer containing the protease inhibitor cocktail (*Roche#04693*-*159001*). The protein bands separated in SDS-PAGE were visualized by Coomassie Brilliant Blue staining, alternatively, signals of the interesting proteins were developed using the specific antibodies and chemiluminescence reagents. The mouse anti-GFP Tag (7G9) antibody (*Abmart#M20004*) was utilized to detect EGFP and/or EGFP-labelled protein, while the mouse anti-α-Tubulin antibody (*SIGMA#T6199*) was used to detect α-tubulin protein in lysates to normalize loading.

### Autophagy assay

Autophagic activity can be assessed by quantifying the assembled autophagosome in cytoplasm or by monitoring the fusion of autophagosome with the lysosome or vacuole. Atg8p (homologous to LC3 in mammals), localized on the autophagosomal membranes, is widely regarded as a biomarker for autophagy [22–24]. By labelling the endogenous Atg8p with an EGFP-tag on its amino-terminal, the fused EGFP-Atg8p will functionally embed into the autophagosomal membranes, and then enter the vacuole with the autophagosomes during autophagy be activated. Due to the resistance of EGFP moiety against the vacuolar proteases, the EGFP fragments (proc.EGFP) will accumulate in vacuole while the Atg8p moiety be degraded. Therefore, autophagic activity increases with increasing levels of the proc.EGFP. In this study, we labelled the endogenous Atg8p with an EGFP-tag on its amino-terminal in FIM1- and T1-related engineered strains using the CRISPR/Cas9 system, and the cell lysates were prepared and analyzed by western blot with the anti-GFP antibody as described above.

### Spot assay

The Brefeldin A (BFA), an antagonist against the ER to *cis*-Golgi trafficking, was used in spot assay for comparing the difference of this anterograde vesicular transport between the FIM1 strain and the T1 mutant. The log-phase cells were harvested and adjusted to an OD_600_ of 0.5, and then continuously diluted to 10^−5^ with ddH_2_O. 3 μL of serial dilutions of each strain was spotted onto YPD plates in the absence (vehicle) or in presence of the BFA (10 μg/mL, *Solarbio#B8581*). Growth differences were recorded following incubation at 30 °C for 48 h.

### Statistical analysis

All the *p*-values were calculated using an unpaired two-tailed *Student’s t* test, which was considered to be significant if the value was less than 0.05. The intensities from western blot assay was quantified using the *GeneTools 4.00*. Each assay was done in triplicate at least and the error bars represent the normalized standard deviation of replications.

## Results

### Secretory expression of the heterologous Est1E was improved by irradiation mutagenesis

**Fig. 1:**
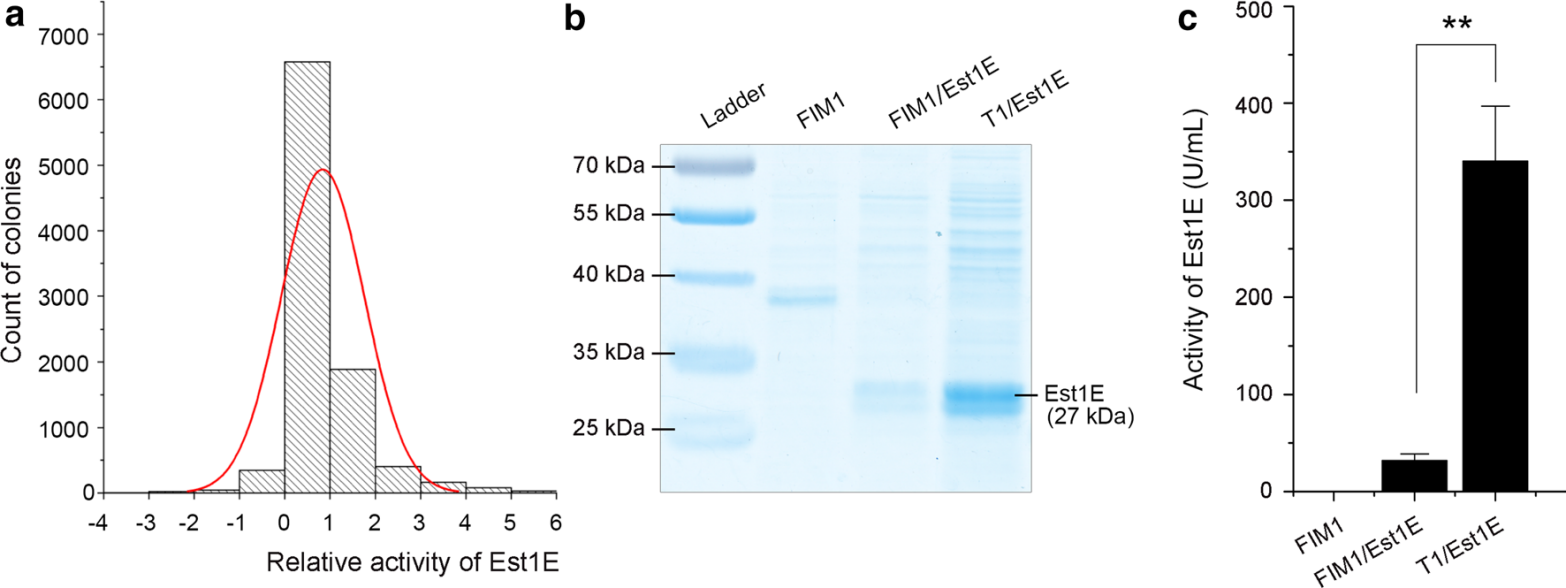
Secretory expression of the heterologous Est1E in *K. marxianus* was improved and identified. **a** Distribution of the relative activity of Est1E secreted by the irradiated mutants against to the wild-type FIM1/Est1E recombinant, the red curve represented normal distribution fitting; **b** SDS-PAGE analysis for evaluating the expression of Est1E in the fermentation supernatants; **c** enzymatic activity of Est1E in the fermentation supernatants; bars ± SD; **p < 0.01 vs FIM1/Est1E recombinant

To evaluate the yields of heterologous protein in *K. marxianus*, a ruminal feruloyl esterase Est1E (*Genbank No. MH212232*, Additional file 1: Table S1) was elected as a biomarker and heterologously expressed in the FIM1 strain or related mutants. As shown in Fig. 1b, SDS-PAGE revealed an additional Est1E brand at 27 kDa in the fermentation supernatant of the FIM1/Est1E. The secreted Est1E exhibited hydrolytic activity on the artificial substrate CNPF with enzyme activity being only 30.46 ± 7.35 U/mL (Fig. 1c).

This FIM1/Est1E recombinant was then used as a parental strain and UV-^60^Co-γ irradiation mutagenesis was carried out to improve the productivity of heterologous proteins. The expression of Est1E secreted by the mutants was calculated by quantifying its hydrolytic activity on CNPF in a high-throughput screening manner (Additional file 1: Figures S1–S3). Statistical analysis showed that the relative activity of Est1E secreted by the mutants against the wild-type followed normal distribution, and only 47 out of the 10,000 mutants showed at least 5-times higher activity of Est1E compared to the parental recombinant (Fig. 1a). Among the 47 mutants, one mutant (termed as T1/Est1E) showed at least tenfold elevated activity of Est1E (322.5 ± 75.08 U/mL) compared to the parental FIM1/Est1E, and was thus identified and employed as a representative for later experiments (Fig. 1b, c). Additionally, under fed-batch fermentation, the Est1E secreted from this T1/Est1E mutant was accumulated in the supernatant up to 1.87 g/L at 72 h (Additional file 1: Figure S4).

### Vesicle trafficking was enhanced while autophagy be weakened in the T1/Est1E mutant

**Fig. 2:**
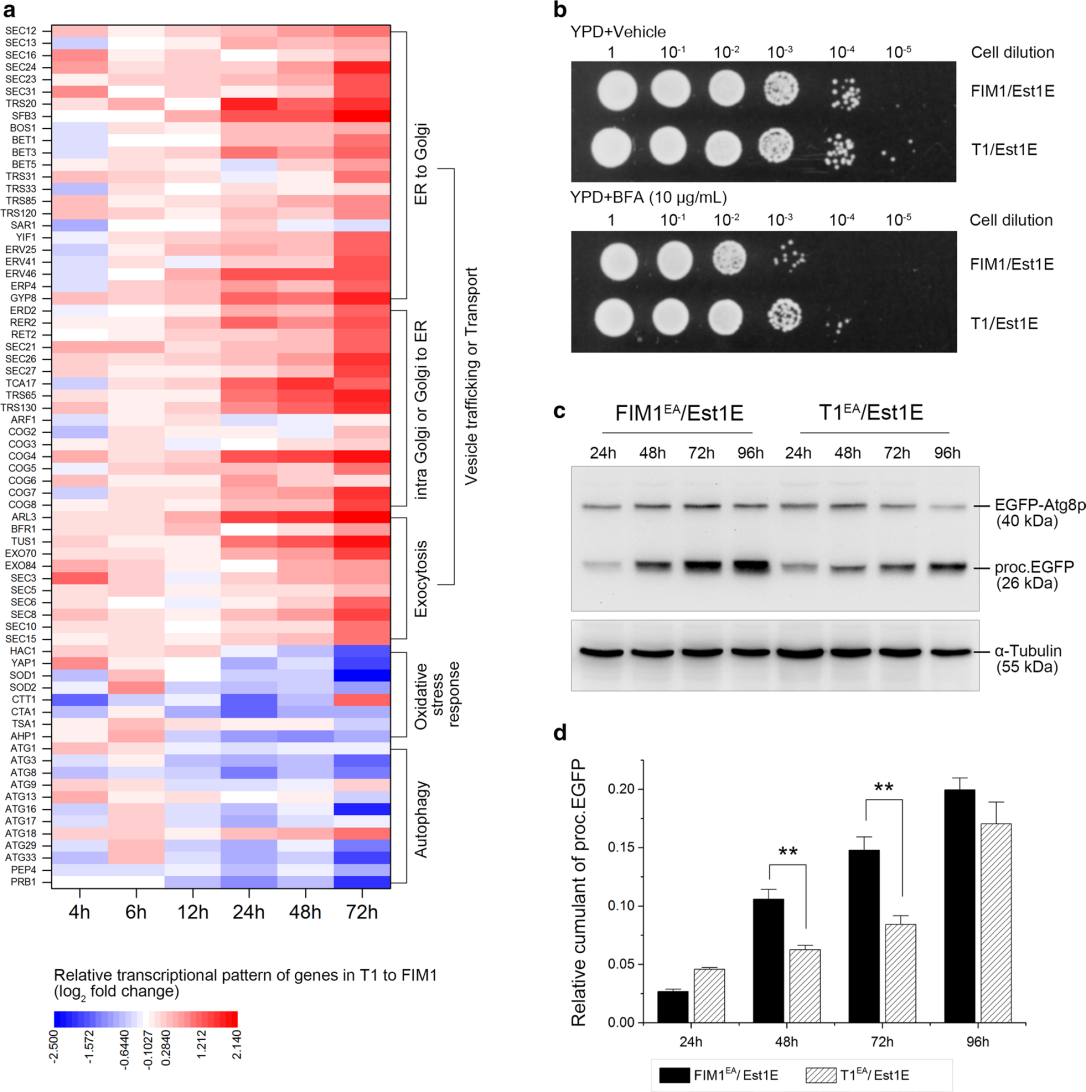
Transcriptomic analysis and experimental verification. **a** Relative transcriptional profile of genes engaged in the intracellular vesicle trafficking, oxidative stress response and autophagy pathways; **b** proliferation and resistance of the FIM1/Est1E or the T1/Est1E recombinants against BFA treatment; **c** Western blot analysis to monitor the cumulant of the vacuolar proc.EGFP fragments, α-tubulin was designed as internal standard; **d** quantitatively analysis for relative cumulant of the proc.EGFP fragments according to its gray intensity of bands showed in **c**, the cumulant of proc.EGFP fragments was normalized with the intensity of its respective α-tubulin; bars ± SD; **p < 0.01 vs FIM1/Est1E recombinant

RNA-seq was performed to track and compare the alteration of genome-wide gene expression in the FIM1/Est1E and the T1/Est1E recombinants (Additional file 2). Transcriptional profiling revealed that a number of genes which were engaged in vesicle trafficking or transport pathways (e.g., *SEC23*, *SEC24*, *TRS20*, *TRS65*, *SFB3* and *ARL3*, etc.) were significantly upregulated in the T1/Est1E mutant in a time-dependent manner, concomitant with noticeable downregulation of many genes related to oxidative stress response (e.g., *HAC1*, *YAP1* and *SOD1*, etc.) and autophagy (e.g., *ATG3*, *ATG8* and *PEP4*, etc.), especially at 72 h over the course of cultivation (Fig. 2a). This inferred that the vesicle trafficking might be intensified while the autophagy might be inhibited in the T1/Est1E recombinant, which was verified in the following experiments.

In the spot assay with 10 μg/mL of BFA, the growth of T1/Est1E recombinant was much better than the FIM1/Est1E under 10^−4^ dilution (Fig. 2b), indicating that the ER to *cis*-Golgi trafficking was enhanced in the T1/Est1E recombinant. To verify the autophagic activity, we quantified the cumulant of the vacuolar proc.EGFP in the indicated recombinants. As shown in Fig. 2c, d, the accumulation of proc.EGFP fragments in both the FIM1/Est1E and the T1/Est1E recombinants increased with time (from 24 to 96 h); in each of the four indicated time-points, the cumulant of proc.EGFP fragments in the FIM1/Est1E was virtually higher than that in the T1/Est1E recombinant (Fig. 2c) and the difference was statistically significant at 48 h and 72 h (*p *< 0.01) (Fig. 2d). These results indicated that autophagy in the T1/Est1E recombinant was lower than that in the FIM1/Est1E, which was consistent with our transcriptional profiling observations.

### Mtc6p, a hypothetical ER protein, played a key role in secretory expressing Est1E in *K. marxianus*

**Fig. 3:**
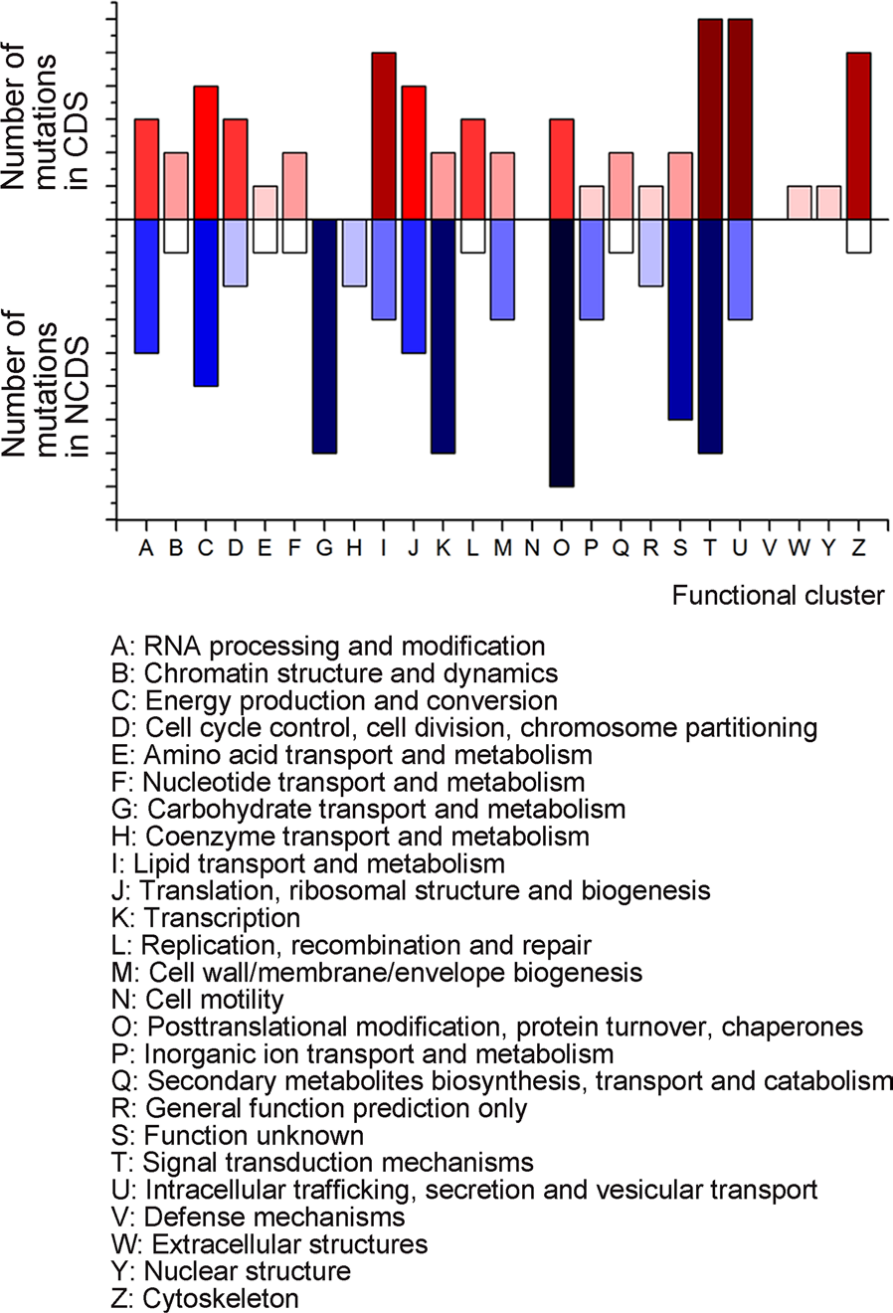
KOG clustering for mutant genes in the T1 mutant

Whole genome sequencing were performed to find out the key genes that might be responsible for the different expression of Est1E between the FIM1/Est1E and the T1/Est1E recombinants. Compared to the FIM1/Est1E, 67 SNPs and 5 indels were found throughout the genome of the T1/Est1E mutant, which led to 31 non-synonymous mutations (Additional file 1: Table S3). Eukaryotic orthologous groups (KOG) analysis showed that 10.2% of the non-synonymous mutant genes were predicted to participate in intracellular trafficking or vesicular transport, 8.47% in regulating cellular cytoskeletal events, and 6.78% in regulating translation or ribosomal biogenesis pathways (Fig. 3).

**Fig. 4:**
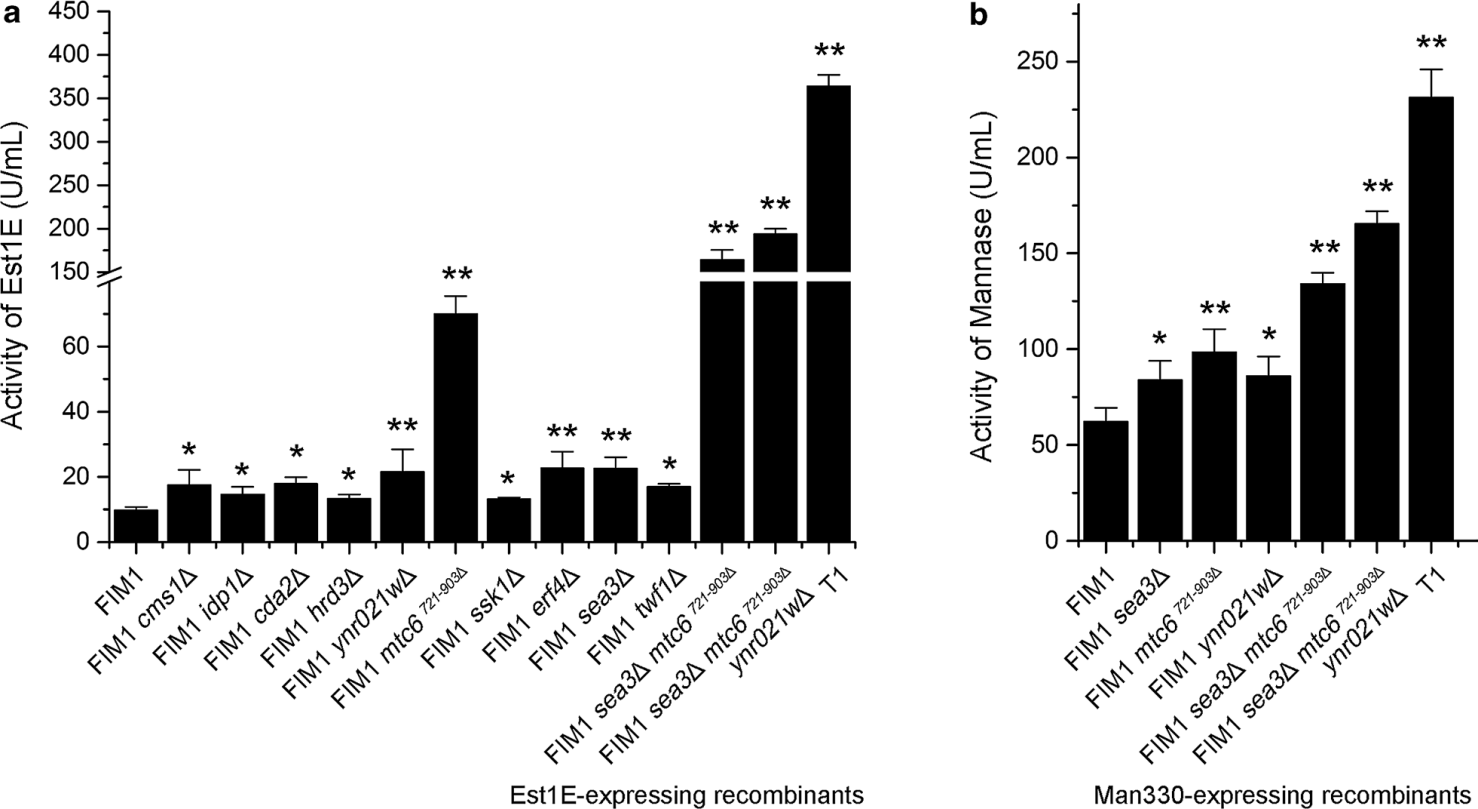
Influences induced by the candidate mutations on secretory expressing the heterologous proteins in *K. marxianus.* **a** Enzymatic activity of Est1E secreted from the indicated mutational Est1E-expressing recombinants; **b** enzymatic activity of mannase secreted from the indicated mutational Man330-expressing recombinants; bars ± SD; *p < 0.05 or **p < 0.01 vs FIM1 recombinant

Considering that the productivity of heterologous Est1E was increased remarkably along with the intracellular vesicle trafficking be enhanced in the T1/Est1E mutant, we only focused on the mutant genes which be predicted to play a role in the amino-acid metabolism (*IDP1*, *PYC2* and *HER2*), the protein biosynthesis (*CMS1*, *MDN1*, *RPF1* and *KRI1*) and the vesicle transport pathways (*HRD3*, *YNR021W*, *MTC6*, *TWF1*, *CAD2*, *ERF4*, *SEA3*, *SSK1*, *DRS2* and *VPS10*). Exclusive of the essential genes, 10 of the interested genes were regarded as candidates and be examined their impact on the productivity of heterologous proteins in *K. marxianus* (Table 2). Gene editing, such as deletion or splicing mutation were accomplished using the CRISPR/Cas9 system (Additional file 1: Figure S5). Unlike other 9 mutations in which we deleted the whole ORF, we only deleted a nucleotide fragment from 721 to 903 of *MTC6* (covering the specific SNP at C755Δ) in FIM1 (FIM1 *mtc6*^721−903Δ^) to disrupt the Mtc6p (coded by *MTC6*) with minimized impact on its adjacent gene, because there was an overlapping region (30 bp) of the 5′-end of *MTC6* with the 5′-end of its adjacent gene, complete deleting the ORF of *MTC6* might have an impact on the expression of this adjacent gene. As shown in Fig. 4, the deletion or splicing of any of the 10 candidates in the FIM1 strain improved the secretory expression of heterologous Est1E (Table 3). Especially, splicing *MTC6* in FIM1 (FIM1 *mtc6*^721–903Δ^/Est1E) resulted in 7.27-fold higher activity of Est1E than that of the FIM1/Est1E (Fig. 4a). Compared to the T1/Est1E which harbored all 31 non-synonymous mutations, splicing *MTC6* contributed 19.22% to the expression of Est1E, while deleting *SEA3* or *YNR021W* only contributed 6.21% or 5.93%, respectively. Notably, double mutation of *mtc6*^721−903Δ^ and *sea3*Δ contributed 45.08% to the expression of Est1E, and triple mutation of *mtc6*^721−903Δ^, *sea3*Δ and *ynr021w*Δ contributed as much as 53.26% (Fig. 4a, Table 3).

**Table 2:** Non-synonymous mutations in the T1 mutant which be involved in amino-acid metabolism, protein biosynthesizing or intracellular trafficking

Genes	Mutations on ORF	Variations	Functional annotation
*CMS1*	A161C	Asp54 to Ala	Putative subunit of the 90S preribosome processome complex
*IDP1*	G1087A	Glu363 to Lys	Mitochondrial NADP-specific isocitrate dehydrogenase
*CDA2*	T391A	Cys131 to Ser	Chitin deacetylase, be involved in cell wall organization
*HRD3*	G526A	Ala176 to Thr	ER membrane protein that plays a central role in ERAD
*YNR021W*	1211+C	Frameshift mutation	ER membrane protein
*MTC6*	C755Δ	Frameshift mutation, Leu256*	Hypothetical ER and vacuolar protein
*SSK1*	A1505C	Asp502 to Ala	Cytoplasmic phosphorelay intermediate osmosensor and regulator
*ERF4*	A789T	Gln262 to His	Palmitoyltransferase subunit
*SEA3*	A1814T	His605 to Leu	Subunit of SEACAT
*TWF1*	A811G	Ile271 to Val	Highly conserved actin monomer-sequestering protein

* Stop codon

**Table 3:** Effect of non-synonymous mutations on the expression of heterologous proteins

Strains	Activity of Est1E (U/mL)	Percentage of Est1E expression in FIM1 mutant over Est1E expression in T1 (%)	Activity of Man330 (U/mL)	Percentage of Man330 expression in FIM1 mutant over Man330 expression in T1 (%)
T1	363.91 ± 12.99	100	231.29 ± 14.64	100
FIM1 *sea3*Δ *mtc6*^721−903Δ^ *ynr021w*Δ	193.81 ± 6.09	53.26	165.39 ± 6.47	71.5
FIM1 *sea3*Δ *mtc6*^721−903Δ^	164.05 ± 11.45	45.08	134.07 ± 5.79	57.97
FIM1 *mtc6*^721−903Δ^	69.95 ± 5.51	19.22	98.52 ± 11.86	42.6
FIM1 *sea3*Δ	22.61 ± 3.39	6.21	83.91 ± 9.97	36.28
FIM1 *ynr021w*Δ	21.57 ± 6.91	5.93	86.05 ± 10.07	37.2
FIM1 *erf4*Δ	22.71 ± 5.03	6.24	Non-detected	–
FIM1 *cda2*Δ	17.91 ± 1.98	4.92	Non-detected	–
FIM1 *twf1*Δ	16.98 ± 0.93	4.67	Non-detected	–
FIM1 *cms1*Δ	17.53 ± 4.63	4.82	Non-detected	–
FIM1 *idp1*Δ	14.63 ± 2.38	4.02	Non-detected	–
FIM1 *hrd3*Δ	13.35 ± 1.24	3.67	Non-detected	–
FIM1 *ssk1*Δ	13.24 ± 0.47	3.64	Non-detected	–

In addition, we also examined the impact of *mtc6*^721−903Δ^*, sea3*Δ and/or *ynr021w*Δ mutations on expressing another heterologous protein in *K. marxianus*, mannase Man330. Compared to T1/Man330, splicing *MTC6* contributed 42.6% to the expression of Man330, while deletion of *SEA3* or *YNR021W* contributed 36.28% or 37.2%, respectively. Furthermore, triple mutation of *mtc6*^721−903Δ^, *sea3*Δ and *ynr021w*Δ contributed as much as 71.5% to the expression of Man330 (Fig. 4b, Table 3). These results indicated that *mtc6*^721−903Δ^, missing the 721–903th nucleotides fragment which covered the SNP at C755, played a key role in the secretory expression of heterologous proteins in *K. marxianus*.

### Premature termination of Mtc6p resulted in increased productivity of Est1E in *K. marxianus*

**Fig. 5:**
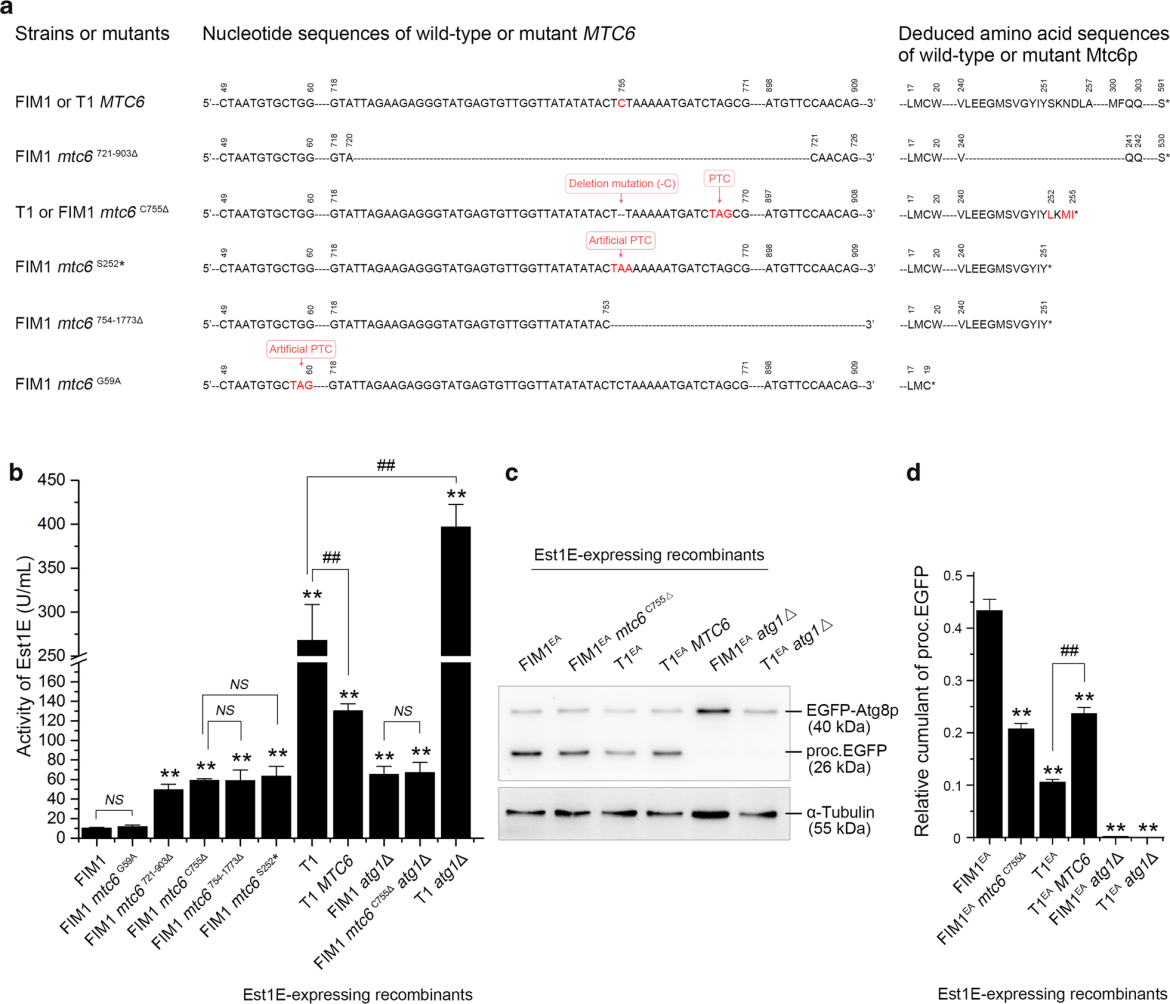
Evaluation of the relationship between Mtc6p and autophagy. **a** Alignment of the nucleotide sequences and deduced amino acid sequences between the wild-type and the mutational *MTC6*; PTC was abbreviated from premature termination codon; **b** enzymatic activity of Est1E expressed and secreted by the indicated recombinants with/without specific mutations on the ORF of *MTC6* or deletion of *ATG1*; **c** cumulant of the vacuolar proc.EGFP fragments in the indicated lysates, α-tubulin was designed as internal standard; **d** quantitatively analysis for relative cumulant of the proc.EGFP fragments according to its gray intensity of bands showed in **c**; bars ± SD; **p < 0.01 vs FIM1/Est1E recombinant, ^##^*p *< 0.01 vs T1/Est1E recombinant; *NS* no statistical significance

Alignment analysis revealed a cytosine deletion on the 755 loci at the ORF of *MTC6* in the T1 mutant, which would otherwise lead to frameshift mutation and translate into a premature polypeptide of 255 amino-acid residues (missing 336 amino-acids on its carboxyl terminus) (Fig. 5a). To verify whether the single-base deletion at C755 in *MTC6* brought the positive influence on expressing the desired proteins, we reinstated the cytosine deletion at 755 loci of *MTC6* in the T1 mutant via CRISPR/Cas9 system and obtained the T1 *MTC6* mutant (Fig. 5a). As shown in Fig. 5b, the productivity of Est1E in the T1 *MTC6*/Est1E recombinant was only 130.28 ± 7.34 U/mL, as compared to 383.93 ± 94.04 U/mL in the T1/Est1E, suggesting that this single-base deletion at C755 of *MTC6* might be responsible for the apparent increase in the yields of desired proteins expressed in *K. marxianus*.

Furthermore, four versions of *MTC6* were constructed in the FIM1 strain using CRISPR/Cas9 system (Fig. 5a, Additional file 1: Figure S5 and Table S1). As shown in Fig. 5a, the FIM1 *mtc6*^C755Δ^ mutant was constructed by deleting a single cytosine at 755 loci of *MTC6* in FIM1 to mimic the aberrant Mtc6p as observed in the T1 mutant, in which frameshift started after Tyr251 and terminated at Ile255 of polypeptide; the FIM1 *mtc6*^S252^* mutant, in which the Ser252 of Mtc6p was substituted into an artificial stop codon and be early terminated at Tyr251; as well as the FIM1 *mtc6*^754−1773Δ^ mutant, which suffered nucleotide deficiency from 754 to 1773 in the ORF of *MTC6* and engendered the same premature polypeptide of 251 amino-acids as the FIM1 *mtc6*^S252^* mutant. These three mutants shared the same sequence of amino-acids from the Met1 to Tyr251, which was identical to the aberrant Mtc6p in T1 mutant. Enzymatic assay indicated that the yields of heterologous Est1E expressed in each of these three mutants were at least 4.86-times higher than that in the FIM1/Est1E recombinant, although no statistical differences be existed between these three recombinants (Fig. 5b). In addition, given that the ORF of *MTC6* overlapped with its adjacent gene, we constructed a FIM1 *mtc6*^G59A^ mutant by substituting the Trp20 of Mtc6p into a stop codon, which would generate a negligible oligopeptide with only 19 amino-acids and be employed to mimic the complete deficiency of Mtc6p (Fig. 5a). However, the productivity of Est1E in the FIM1 *mtc6*^G59A^/Est1E recombinant was not significantly higher than that in the FIM1/Est1E (Fig. 5b). These suggested that the premature termination of Mtc6p, be induced by the single-base deletion at C755 in ORF, enabled the recombinant to efficiently express and secrete the heterologous proteins in *K. marxianus*.

### Premature termination of Mtc6p resulted in attenuated autophagy in *K. marxianus*

Since the results from Fig. 2c showed that autophagy was inhibited in the T1/Est1E recombinant while displayed increased productivity of Est1E, we investigated whether the Mtc6p was involved in autophagy. As shown in Fig. 5c, d, the cells failed to process and produce proc.EGFP fragment when its endogenous *ATG1* be deleted, indicating that the cellular autophagy should be complete interdicted by deleting *ATG1* in *K. marxianus*, and these *atg1*Δ mutants could be regarded as a control. Compared to FIM1^EA^ which harbored the wild-type Mtc6p, the cumulant of proc.EGFP fragment was significantly decreased in the FIM1^EA^ *mtc6*^C755Δ^; whereas the accumulation of proc.EGFP fragment was dramatically increased in the T1^EA^ *MTC6* mutant with reinstated Mtc6p, as compared to the T1^EA^ mutant with an intrinsic premature Mtc6p (*mtc6*^C755Δ^) (Fig. 5c, d). These results indicated that the single-base deletion of C755 in *MTC6* leaded to incomplete interdiction of autophagy, suggesting that Mtc6p might be involved in regulating autophagy in *K. marxianus*.

We then examined the productivity of Est1E in the *atg1*Δ mutant, in which autophagy was interdicted. As shown in Fig. 5b, compared to FIM1/Est1E, the expression of Est1E was dramatically increased in the FIM1 *atg1*Δ/Est1E recombinant; and the expression of Est1E in the FIM1 *mtc6*^C755Δ^ *atg1*Δ/Est1E recombinant was slightly higher than that in the FIM1 *mtc6*^C755Δ^/Est1E, albeit no significant difference of the productivity of Est1E existed between the FIM1 *atg1*Δ/Est1E and the FIM1 *mtc6*^C755Δ^ *atg1*Δ/Est1E recombinants. Moreover, compared to T1/Est1E, the productivity of Est1E substantially increased in the T1 *atg1*Δ/Est1E recombinant. These results suggested that attenuating autophagy would improve the yields of desired proteins expressed in *K. marxianus*.

### Yields of various heterologous proteins could be improved by attenuating autophagy

**Fig. 6:**
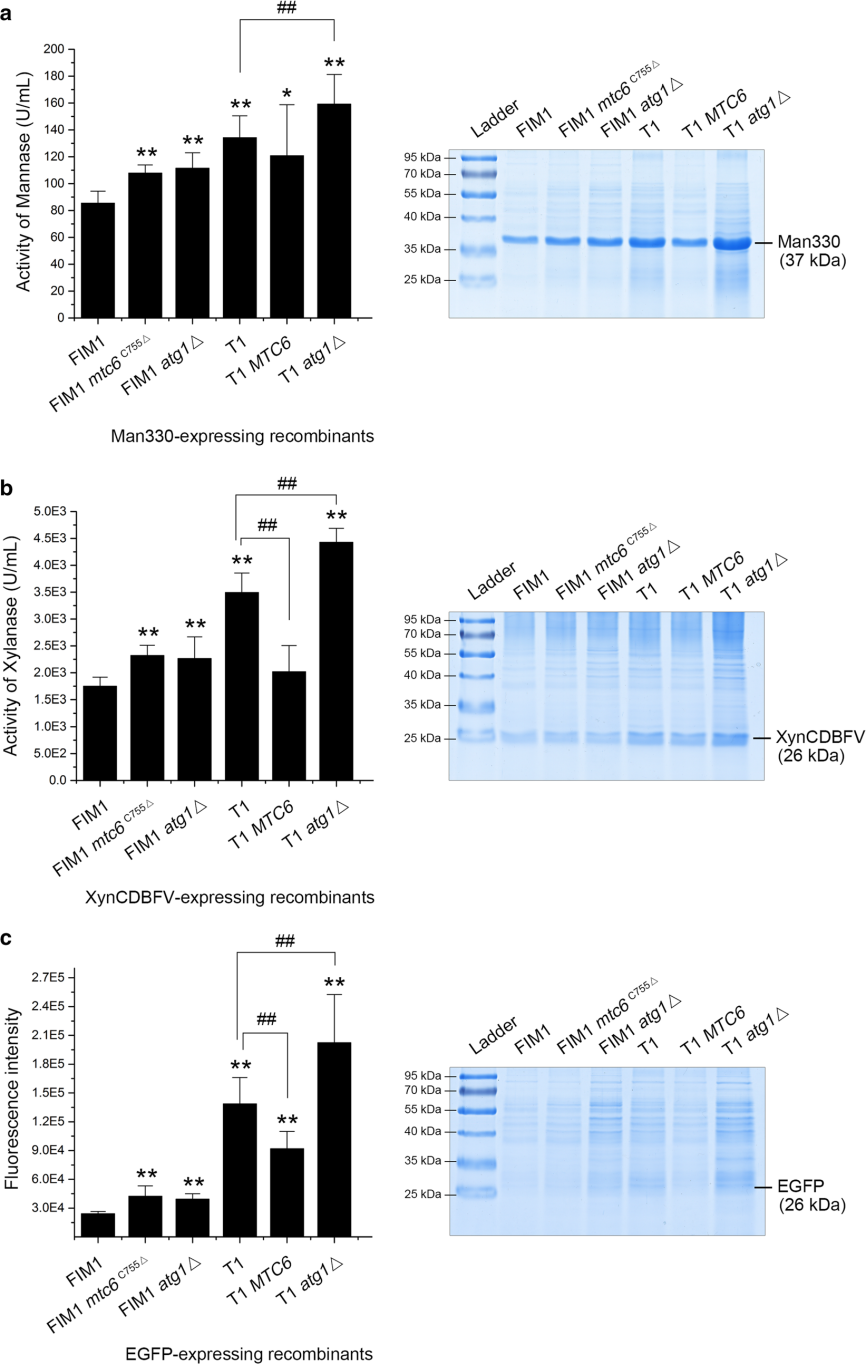
Influences of attenuated or interdicted autophagy on secretory expression of the heterologous proteins in *K. marxianus.* **a** Enzymatic activity and expression of the mannase Man330 in the indicated fermentation supernatants; **b** enzymatic activity and expression of the β-1,4-endoxylanase XynCDBFV in the indicated fermentation supernatants; **c** fluorescence intensity and expression of EGFP in the indicated fermentation supernatants; bars ± SD; *p < 0.05 or **p < 0.01 vs the FIM1 recombinant, ^##^*p *< 0.01 vs the T1 recombinant

Extensively, in order to evaluate the impact of attenuated autophagy on expressing the desired proteins in *K. marxianus*, other heterologous proteins, such as the mannase Man330 (~ 37 kDa), the β-1,4-endoxylanase XynCDBFV (~ 26 kDa, glycosylated) or the conventional EGFP (~ 26 kDa), was respectively expressed in the FIM1 strain or the indicated mutant which harbored mutational Mtc6p (i.e., the FIM1 *mtc6*^C755Δ^ and T1) or deficient Atg1p (i.e., the FIM1 *atg1*Δ and T1 *atg1*Δ). As shown in Fig. 6, the secretory expression of all these three proteins was significantly increased in the recombinants with *mtc6*^C755Δ^ (i.e., FIM1 *mtc6*^C755Δ^ and T1) or *atg1*Δ than that in the recombinants with wild-type *MTC6* (i.e., FIM1 and T1 *MTC6*). Parallel to enzymatic activity or autofluorescence analyses, SDS-PAGE analysis confirmed observable enhancement in secretory expression of these desired proteins in context (Fig. 6). These observations indicated that the yields of various heterologous proteins expressed in *K. marxianus* could be improved by inhibiting or interdicting autophagy, and the *Mtc6p* might be designed as a potential target for modulating the native autophagy.

## Discussion

In this work, we identified one *K. marxianus* mutant (termed T1) with the ability to efficiently express and secrete various desired proteins, whatever the heterologous ruminal feruloyl esterase Est1E, the mannase Man330, the β-1,4-endoxylanase XynCDBFV or the EGFP. The T1/Est1E recombinant yielded 1.87 g/L of Est1E in supernatant under fed-batch fermentation for 72 h, to our knowledge, it was so far the highest reported yields of heterologous protein produced by *K. marxianus*. It meant that this T1 mutant held the potential to be designed as chassis and be used in the fundamental experiments or the industrial applications.

Compared to the parental FIM1/Est1E recombinant, the vesicle trafficking was enhanced while autophagy be weakened in the T1/Est1E mutant. It might be explained by the fact that the upregulated Trs65p (Fig. 2a), a subunit of the TRAPPII complex which participates in an Arf1p-GEF effector loop [25], would facilitate TRAPPII to interact with Arf1p and boost COPI-mediated vesicle trafficking, in turn enable the T1 mutant to withstand BFA cytotoxicity (Fig. 2b). Besides, the intensive COPI-vesicles would effectively retrieve the escaped luminal proteins or components which be required for anterograde transport, this benefits the COPII-vesicles assembling and would facilitate the vesicles to carry cargoes from ER to *cis*-Golgi [26]. The intensive circuit between the ER and the Golgi apparatus would not only stabilize the ER–Golgi interface, but also lighten the ER-stress, and then inactivate the unfolded protein response (UPR) and the ER-associated degradation (ERAD) pathway [27]. In this case, the feedback inhibition induced by UPR on transcriptional activity of the target genes would be repealed, and more precursor proteins would escape being degraded from the ERAD pathway.

Moreover, we identified and reported a novel functional protein Mtc6p, that was the premature Mtc6p, which was induced by single-base deletion at C755 in its ORF and be early terminated at Tyr251 of polypeptide, dramatically improved the secretory expression of desired proteins, accompanying with attenuated autophagy. And the yields of desired proteins was further improved by interdicting autophagy, although no more improvement be detected in the double mutant with truncated Mtc6p and deficient Atg1p (FIM1 *mtc6*^C755Δ^ *atg1*Δ) as compared to the FIM1 *atg1*Δ mutant (Figs. 2, 5). This inferred that the Mtc6p might be a downstream target of Atg1p and be involved in regulating autophagy in *K. marxianus*. Intriguingly, complete lack of Mtc6p in FIM1 (FIM1 *mtc6*^G59A^) failed to elevate the productivity of Est1E in *K. marxianus*, while only the carboxyl-terminal truncated Mtc6p did (i.e., the FIM1 *mtc6*^C755Δ^, FIM1 *mtc6*^S252^* and FIM1 *mtc6*^754−1773Δ^). It could be supposed as the negative dominant effect, in which the carboxyl-terminal truncated Mtc6p might inhibit the function of other molecules by adversely interacting. However, the specific molecular mechanism and their spatiotemporal interaction required for further exploration.

So far as we know, it was the first report that autophagy brought negative influence on biosynthesizing the desired proteins in the engineered yeasts, especially in *K. marxianus*. Previous studies revealed that the secretory and autophagy pathways are intimately linked and shared many machineries, although they are generally thought of as a biosynthetic or a degradative branch of the endomembrane system [16, 28]. For instance, Atg1p not only phosphorylates Atg9p for organizing the autophagosome, but also phosphorylates the Sec23Ap at Ser207 and Thr405 residues, which otherwise reduce the interaction between Sec23Ap and Sec31Ap, eventually suppress the secretion routes [29, 30]. The ERES provides biomembranes or some essential components for assembling the autophagosome through COPII-coated vesicles, which could assist the phagophore in building or maturation into an autophagosome [16, 28]. All these evidence implied a tradeoff existed between the autophagy and vesicular secretion, which led to both the pathways to contend for constituent or functional elements. As we demonstrated in this manuscript, repressing the cellular autophagy improved the yields of various heterologous proteins in *K. marxianus*, it might be explained that the essential resources were reallocated from the attenuated autophagy to the secretory pathway, what intensified its vesicle trafficking and then improved the secretory expression of the desired proteins (Figs. 2, 5).

Based on these findings, we raised a prospect that whether the yields of desired proteins will be higher if the engineered yeast be modified by attenuating autophagy coupled with the existing strategies, and much more efforts are undergoing to optimize these microbial cell factories for biosynthesizing the desired proteins.

## Conclusions

This was the first study to report that the Mtc6p be involved in regulating autophagy in *K. marxianus*, and that inhibited or interdicted autophagy could lead to a substantial increase in the yields of desired proteins in *K. marxianus*. In view of this, the Mtc6p could regarded as a potential target for modulating autophagy, although further studies are warranted to investigate the molecular mechanisms of Mtc6p in regulating autophagy.

## Additional files

**Additional file 1: Figure S1.** Strategies for screening desirable mutants in a high-throughput manner. **Figure S2.** A linear relationship was existed between the activity of Est1E and the enzyme concentration. **Figure S3.** Identification of the mutants with excellent ability in expressing the heterologous Est1E. **Figure S4.** Purification and quantification of the Est1E expressed and secreted by the T1/Est1E recombinant. **Figure S5.** Establishment of the specific mutants using CRISPR/Cas9 system. **Table S1.** Amino-acid sequences of the Est1E and the full or truncated Mtc6p. **Table S2.** Primers and DNA sequences used in this study. **Table S3.** Information of the specific SNP or indel in the genome of T1 mutant.

**Additional file 2.** Detail information of the transcriptional analysis.

## Abbreviations

BFA: Brefeldin A
CNPF: 2-chloro-4-nitropheyl ferulate
EGFP: enhanced green fluorescent protein
ER: endoplasmic reticulum
ERES: endoplasmic reticulum exit sites
ERAD: ER-associated degradation
GOX: glucose oxidase
indel: insertion or deletion of oligonucleotide fragments
KOG: eukaryotic orthologous groups
ORF: open reading frame
PTC: premature termination codon
SNP: single nucleotide polymorphisms
SOD: superoxide dismutase
UPR: unfolded protein response
