# Supplementary material for: Mutational Mtc6p attenuates autophagy and improves secretory expression of heterologous proteins in Kluyveromyces marxianus
Source: Microb Cell Fact. 2018 Sep 14;17:144. doi: 10.1186/s12934-018-0993-9 (PMC6138896; doi:10.1186/s12934-018-0993-9)
Supplement: Supplementary file 1 — Additional file 1: Figure S1. Strategies for screening desirable mutants in a high-throughput manner. Figure S2. A linear relationship was existed between the activity of Est1E and the enzyme concentration. Figure S3. Identification of the mutants with excellent ability in expressing the heterologous Est1E. Figure S4. Purification and quantification of the Est1E expressed and secreted by the T1/Est1E recombinant. Figure S5. Establishment of the specific mutants using CRISPR/Cas9 system. Table S1. Amino-acid sequences of the Est1E and the full or truncated Mtc6p. Table S2. Primers and DNA sequences used in this study. Table S3. Information of the specific SNP or indel in the genome of T1 mutant. [file 12934_2018_993_MOESM1_ESM.docx]

**Additional file 1:**


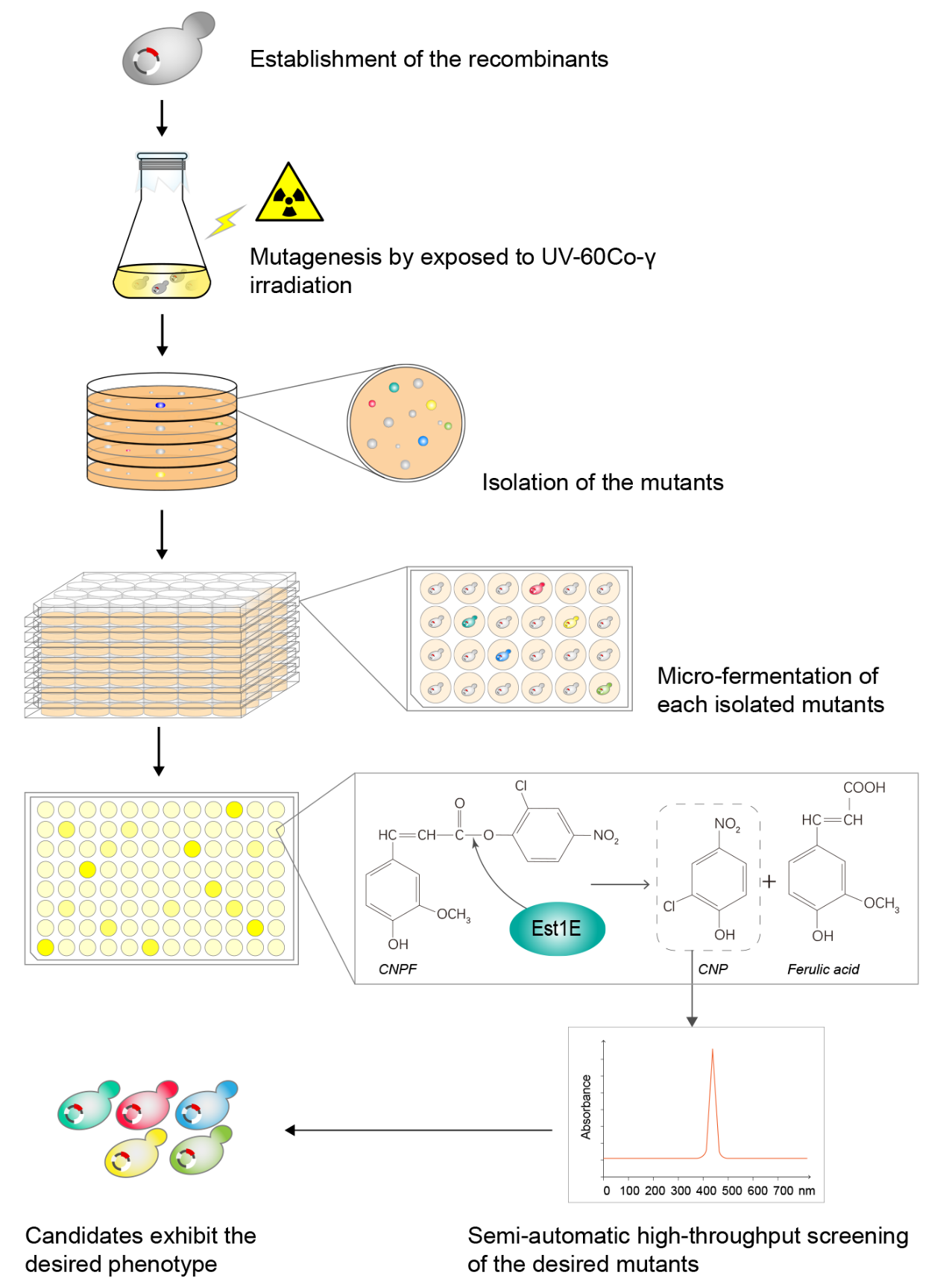


**Fig. S1** **Strategies for screening desirable mutants in a high-throughput manner**


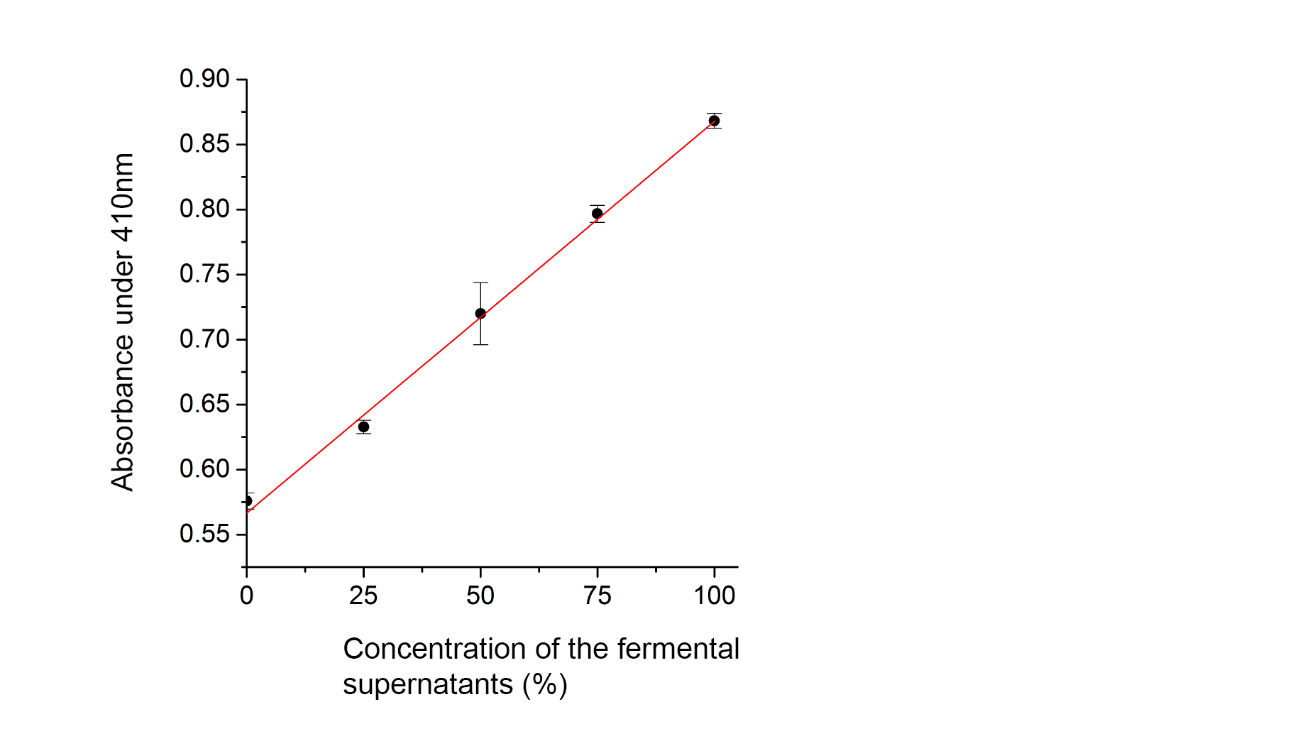


**Fig. S2 A linear relationship was existed between the activity of Est1E and the enzyme concentration**

In this work, the value of absorbance under 410nm reflected the specific activity of Est1E in the reaction, and the crude enzyme (fermentation supernatants) was diluted continuously with 1×PBST buffer to diverse concentration. The red line was a fitted line that indicated a linear relationship be existed between the activity of Est1E and the enzyme concentration. Bars, ±SD.


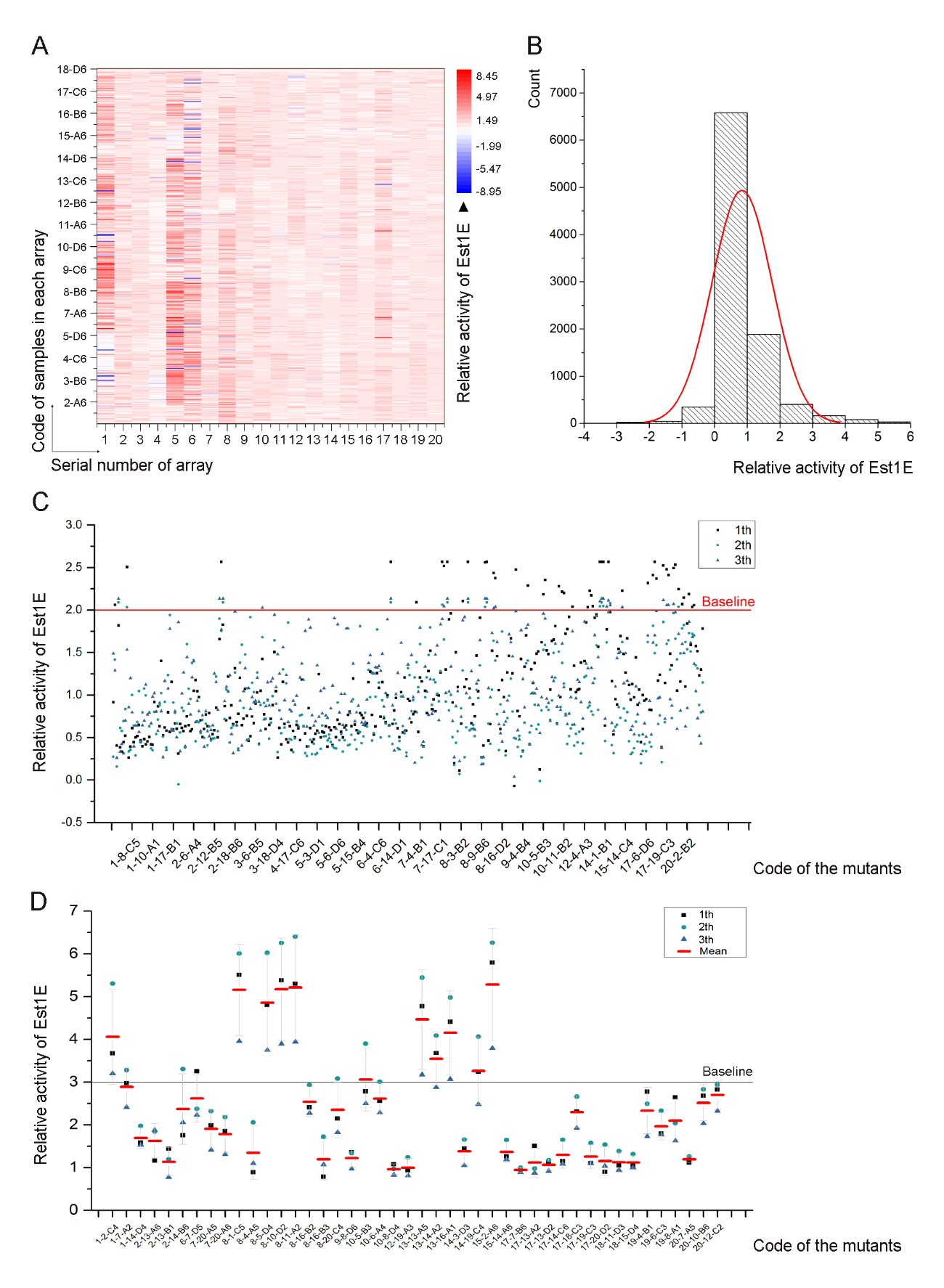


**Fig. S3 Identification of the mutants with excellent ability in expressing the heterologous Est1E**

1. Ⅰ-phase high-throughput screening from 10 000 mutational colonies after UV-^60^Co-γ-ray irradiation mutagenesis;
2. Normal distribution curve of the 10 000 samples under the Ⅰ-phase high-throughput screening for efficient expression strains;
3. Ⅱ-phase high-throughput screening for efficient expression mutants from the 384 candidates derived fromⅠ-phase;
4. Ⅲ-phase screening for the mutants with tripled expression pattern at least from the 44 candidates derived from the aforementioned Ⅱ-phase screening.

***Fermentation, purification and quantification of Est1E***

Fed-batch cultivation was carried out in a fermentor with a working volume of 5L at 30 °C. YPD medium was used as basic medium, and 1000 g/L of Glucose solution was used as the feed medium, in which the amount of medium added in the fed-batch phase was recorded by continuous monitoring the weights of the reservoir vessels and the fermentor. The pH was controlled automatically at 6.5 using 7.5M Ammonium hydroxide,

aeration was automatically adjusted to keep the dissolved-O_2_ tension (DOT) above 50% air saturation at a constant stirrer speed of 800 rpm and the pressure was adjusted manually up to a maximum of 2.5 bar absolute pressure.

The fermentation supernatant was collected and filtrated before purification by 0.45 μm syringe filters, and adjusted the pH value to 6.5 with 1M HCl. The sample was loaded onto a *HiTrap^TM^ Q-FF* column (*GE Healthcare#17-6002-33*), which had been equilibrated in 10 mM Bis-Tris (pH6.5), and the column was washed with 10 mM Bis-Tris (pH6.5) and 5-10% 1M NaCl. Est1E was eluted from the column with 10 mM Bis-Tris (pH6.5) and 10-50% 1M NaCl, and fractions (2 mL) were collected throughout the linear gradient elution and analyzed by SDS-PAGE prior to further purification by size-exclusion chromatography. Molecular sieve purification was carried out using a *Superdex^TM^ 200 Increase 10/300GL* column (*GE Healthcare#28-9909-44*) equilibrated in 20 mM Tris-HCl (pH7.4) with 150mM NaCl using the AKTA purification system (*GE Healthcare*). And then, fractions were collected for SDS-PAGE and enzymatic activity analyses. Protein concentration was estimated by the acknowledged Bradford method, from which the specific activity of Est1E would be calculated. In this case, the secreted Est1E was purified and its specific activity was determined to be 343 U/mg (Fig.S4 A/B).

The T1/Est1E recombinant was submitted for fed-batch fermentation, the Est1E secreted from this engineered recombinant was accumulated in the supernatant, and the highest production was determined at 72 h with enzymatic activity of 641 U/mL, approximating to 1.87 g/L of Est1E recombinant protein produced in the supernatant (Fig.S4 C).


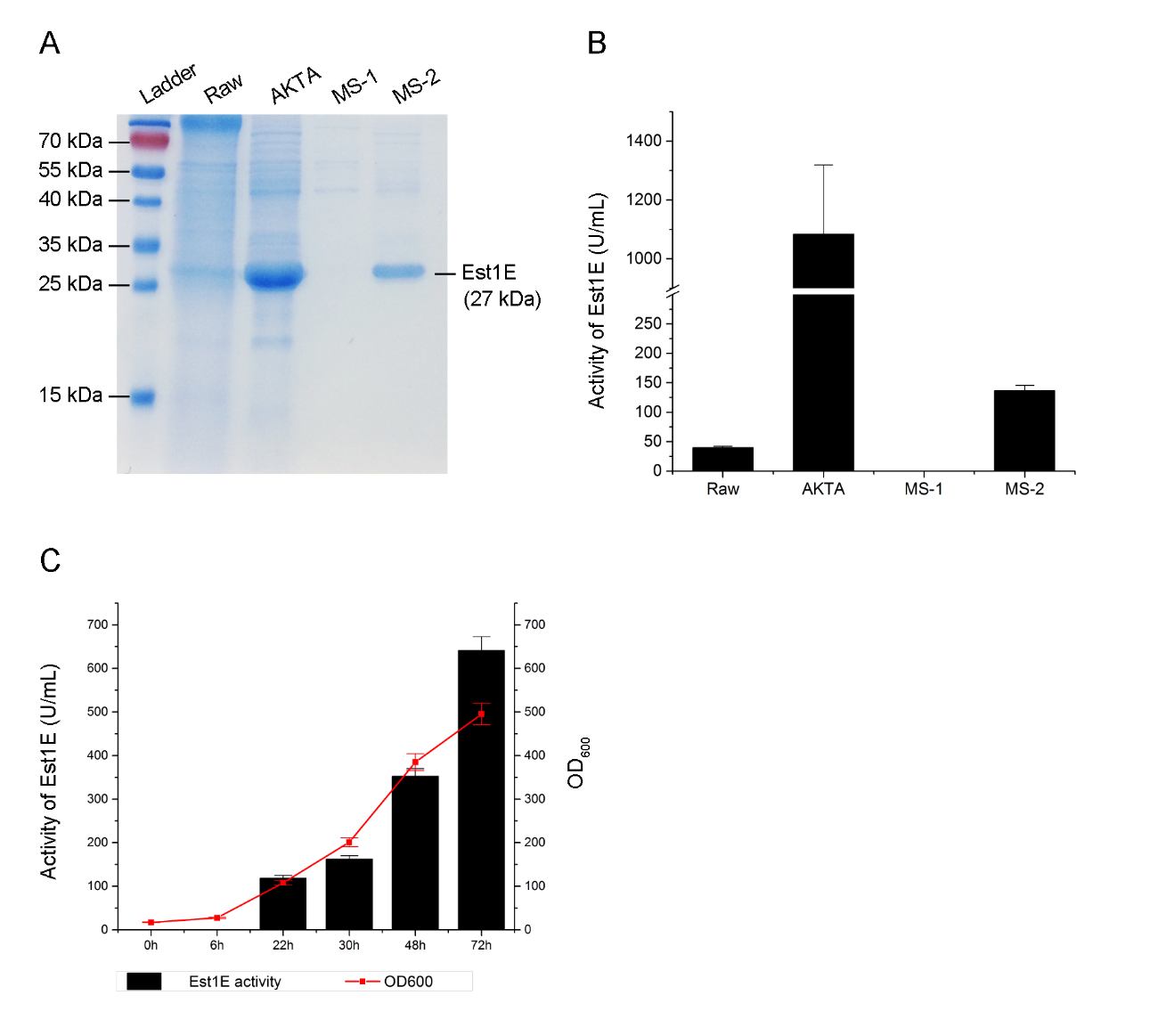


**Fig. S4 Purification and quantification of the Est1E expressed and secreted by the T1/Est1E recombinant**

1. SDS-PAGE analysis to assess the fineness of the purified Est1E followed by ion-exchange chromatography and size-exclusion chromatography;
2. Enzymatic activity of Est1E in the indicated samples;
3. Enzymatic activity of Est1E and OD_600_ of the cultures of T1/Est1E under fed-batch fermentation. Bars, ±SD.


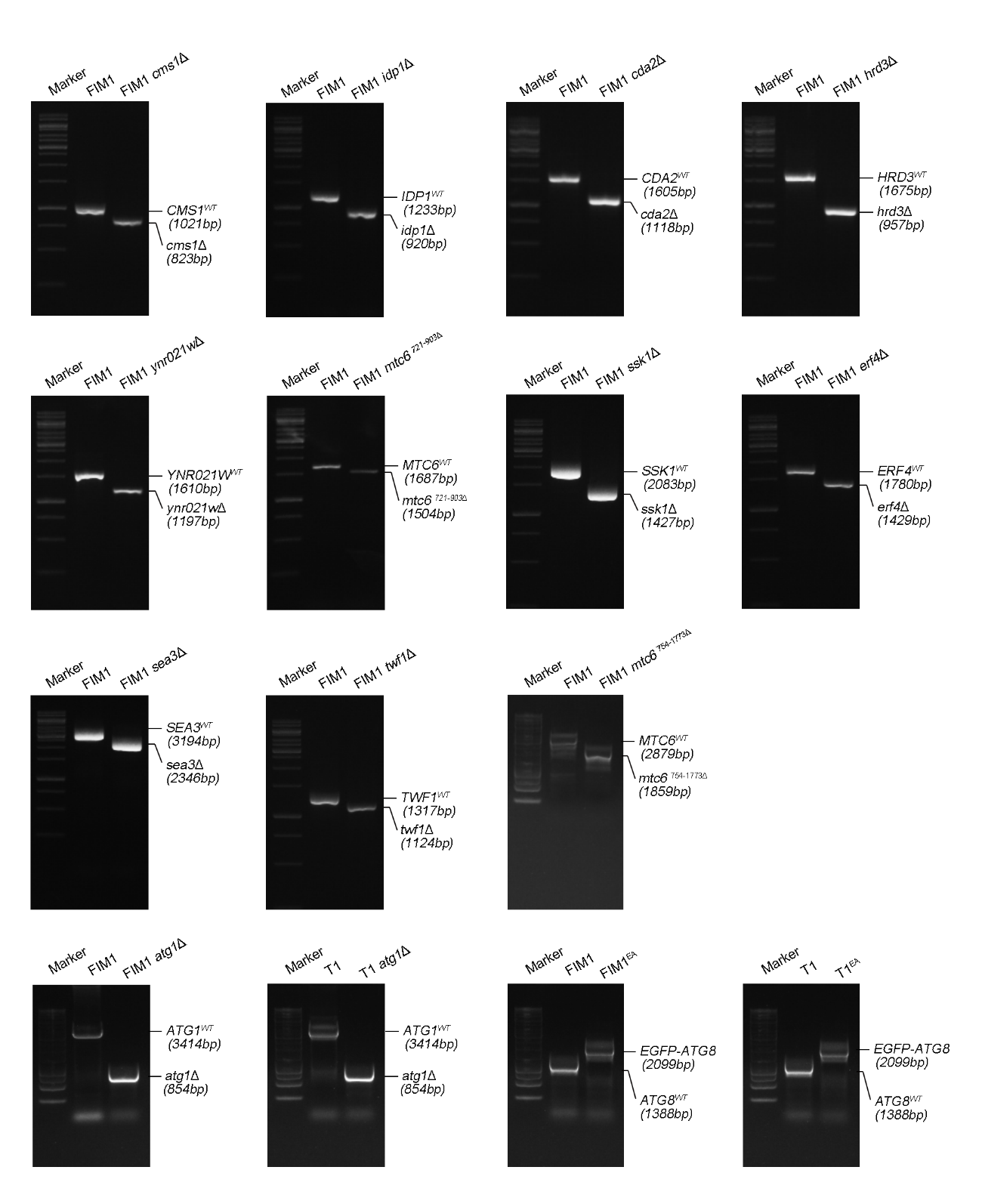


**Fig. S5 Establishment of the specific mutants using CRISPR/Cas9 system**

**Table S1 Amino-acid sequences of the Est1E and the full or truncated Mtc6p**

| **Proteins or polypeptides** | **Amino-acid sequences** |
| --- | --- |
| Est1E | MYIDCDGIKLNAYLDMPKNNPEKCPLCIIIHGFTGHSEERHIVAVQETLNEIGVATLRADMYGHGKSDGKFEDHTLFKWLTNILAVVDYAKKLDFVTDIYMAGHSQGGLSVMLAAAMERDIIKALIPLSPAAMIPEIARTGELLGLKFDPENIPDELEAWDGRKLKGNYARVAQTIRVEDFVDKYQKPVLIVHGDQDEAVPYEFSVKFSKQYKNCKLVTIPGDTHCYDHHLELVTEAVKEFMLEQIAK |
| Wide-type Mtc6p  (in the FIM1 strain or the T1 *MTC6* mutant) | MAVLALMVAHLGMYDLLMCWLSCVCFVTTFHRKKFIVHRSKVSIPAFIYRNSFNMRKIDEVVRLAYNSKRYPGLWELSVTVNGGMVLTLLRYLCFWAFFGVSLAQEWPPFSNEMINALRSQRDLMTNVSIDQIPFPGISIKPALSQSNTLNQTAYLETISELLNHGIQTFQIDLEFDTSSQDWFLEDTGTRFVDVLSTINEYLGVSNTDLNANLISILIRFNNDTLKKSNLFKNSNFTSVLEEGMSVGYIYSKNDLANDRALNQTWDINGYSKDGWVSLNRFLYVVKRRVVFGFLNGDDMFQQDDNPLVFPSETFHYVTEEGTLQCPLKTMDDIGEMSKKQWRFLEGNFTYRNFLQYIECGYSLILTNPVQRSNLSQENEFQRRLTSLLLWSWNATTPDDILDADEDDANSNSQYVAYRCGVFTYTEHEYLAPFKIGNCYRSMPYLCRYSDRAYVWNISEEQGTYFDSEKDHVCPGDHQFGIPRNPLQQRSIRIYLEEEGFDEKDFWIDINSISVKNCWISGGTYASCPYQRYGSTSNYLAMIIPSALIAFFLLLVMFYFNWAHIPIQDNRNNWKRIITAYSKEEVEGVPS |
| Spliced Mtc6p  (in the FIM1 *mtc6*^721-903Δ^ mutant) | MAVLALMVAHLGMYDLLMCWLSCVCFVTTFHRKKFIVHRSKVSIPAFIYRNSFNMRKIDEVVRLAYNSKRYPGLWELSVTVNGGMVLTLLRYLCFWAFFGVSLAQEWPPFSNEMINALRSQRDLMTNVSIDQIPFPGISIKPALSQSNTLNQTAYLETISELLNHGIQTFQIDLEFDTSSQDWFLEDTGTRFVDVLSTINEYLGVSNTDLNANLISILIRFNNDTLKKSNLFKNSNFTSVQQDDNPLVFPSETFHYVTEEGTLQCPLKTMDDIGEMSKKQWRFLEGNFTYRNFLQYIECGYSLILTNPVQRSNLSQENEFQRRLTSLLLWSWNATTPDDILDADEDDANSNSQYVAYRCGVFTYTEHEYLAPFKIGNCYRSMPYLCRYSDRAYVWNISEEQGTYFDSEKDHVCPGDHQFGIPRNPLQQRSIRIYLEEEGFDEKDFWIDINSISVKNCWISGGTYASCPYQRYGSTSNYLAMIIPSALIAFFLLLVMFYFNWAHIPIQDNRNNWKRIITAYSKEEVEGVPS |
| Truncated Mtc6p  (in the FIM1 *mtc6*^C755Δ^ or the T1 mutant) | MAVLALMVAHLGMYDLLMCWLSCVCFVTTFHRKKFIVHRSKVSIPAFIYRNSFNMRKIDEVVRLAYNSKRYPGLWELSVTVNGGMVLTLLRYLCFWAFFGVSLAQEWPPFSNEMINALRSQRDLMTNVSIDQIPFPGISIKPALSQSNTLNQTAYLETISELLNHGIQTFQIDLEFDTSSQDWFLEDTGTRFVDVLSTINEYLGVSNTDLNANLISILIRFNNDTLKKSNLFKNSNFTSVLEEGMSVGYIYLKMI |
| Truncated Mtc6p  (in the FIM1 *mtc6*^S252^* mutant) | MAVLALMVAHLGMYDLLMCWLSCVCFVTTFHRKKFIVHRSKVSIPAFIYRNSFNMRKIDEVVRLAYNSKRYPGLWELSVTVNGGMVLTLLRYLCFWAFFGVSLAQEWPPFSNEMINALRSQRDLMTNVSIDQIPFPGISIKPALSQSNTLNQTAYLETISELLNHGIQTFQIDLEFDTSSQDWFLEDTGTRFVDVLSTINEYLGVSNTDLNANLISILIRFNNDTLKKSNLFKNSNFTSVLEEGMSVGYIY |
| Truncated Mtc6p  (in the FIM1 *mtc6*^754-1773Δ^ mutant) | MAVLALMVAHLGMYDLLMCWLSCVCFVTTFHRKKFIVHRSKVSIPAFIYRNSFNMRKIDEVVRLAYNSKRYPGLWELSVTVNGGMVLTLLRYLCFWAFFGVSLAQEWPPFSNEMINALRSQRDLMTNVSIDQIPFPGISIKPALSQSNTLNQTAYLETISELLNHGIQTFQIDLEFDTSSQDWFLEDTGTRFVDVLSTINEYLGVSNTDLNANLISILIRFNNDTLKKSNLFKNSNFTSVLEEGMSVGYIY |
| Deficient Mtc6p  (in the FIM1 *mtc6*^G59A^ mutant) | MAVLALMVAHLGMYDLLMC |

**Table S2 Primers and DNA sequences used in this study**

| Targets | Applications | Names | Sequences (5’-3’) |
| --- | --- | --- | --- |
| *EST1E* | Amplification of the ORF | N112-Est1E-F | 5'>GAGACGGTGACCCCGGGACTAGTTACATCGACTGTGATGGCATCAAG<3' |
|  |  | N112-Est1E-R | 5'>CAAAGCTTGCGGCCTTAAGCGGCCGCTTACTTAGCGATCTGCTCCAA<3' |
| *EFF4* | Target deletion by CRISPR/Cas9 | 443-PAM1-F | 5’>ATCTCCGTTTGATACATACCGAA<3’ |
|  |  | 443-PAM1-R | 5’>AACTTCGGTATGTATCAAACGGA<3’ |
|  |  | 443-D1uF | 5’>GACTCATCTGCCTTCGGTTTG<3’ |
|  |  | 443-D1uR | 5’>GGCTTCTATTATTTCGGACATACTCGCATCCTTTACATTCAC<3’ |
|  |  | 443-D1dF | 5’>GTGAATGTAAAGGATGCGAGTATGTCCGAAATAATAGAAGCC<3’ |
|  |  | 443-D1dR | 5’>GAAGAGCAAGAGGCGAGGGA<3’ |
|  |  | 443-QC1-F | 5’>CAACAATAGCACCCGCACTACTCCCG<3’ |
|  |  | 443-QC1-R | 5’>GGGGAAAAAGAACAACAGAACTAAAGCC<3’ |
| *CDA2* | Target deletion by CRISPR/Cas9 | 2010-PAM1-F | 5’>ATCTGAGAAATATGAACCATATA<3’ |
|  |  | 2010-PAM1-R | 5’>AACTATATGGTTCATATTTCTCA<3’ |
|  |  | 2010-D1uF | 5’>GGTGCAGAAGGATGAATGG<3’ |
|  |  | 2010-D1uR | 5’>ACCAAGTATGAGTTCCGATGCGGCAGAAACTAAGACTATG<3’ |
|  |  | 2010-D1dF | 5’>CATAGTCTTAGTTTCTGCCGCATCGGAACTCATACTTGGT<3’ |
|  |  | 2010-D1dR | 5’>ATCGTGCTCAAGAATCAAACC<3’ |
|  |  | 2010-QC1-F | 5’>AAAGAAGGGCCAATTAGAATAGCCAAGA<3’ |
|  |  | 2010-QC1-R | 5’>GGTCGGAACCAATAATATCATTTACCTTCAT<3’ |
| *YNR021W* | Target deletion by CRISPR/Cas9 | 2188-PAM1-F | 5’>ATCGAGAAACCAACACTCTGAGA<3’ |
|  |  | 2188-PAM1-R | 5’>AACTCTCAGAGTGTTGGTTTCTC<3’ |
|  |  | 2188-D1uF | 5’>ACAATAAATATGCCGACAAG<3’ |
|  |  | 2188-D1uR | 5’>TTTCCTTACTTCATCGTAGTGATAATACAGCGAGGAGA<3’ |
|  |  | 2188-D1dF | 5’>TCTCCTCGCTGTATTATCACTACGATGAAGTAAGGAAA<3’ |
|  |  | 2188-D1dR | 5’>GCACCTTCTTCAGCTTTAA<3’ |
|  |  | 2188-QC1-F | 5’>ATGTCAAGTATCTGGGGTTCGATTG<3’ |
|  |  | 2188-QC1-R | 5’>GCCTGTGGTAGCAAAGCAACCTCAAC<3’ |
| *SEA3* | Target deletion by CRISPR/Cas9 | 2622-PAM1-F | 5’>ATCCTCGACTATTGAGTCATCAG<3’ |
|  |  | 2622-PAM1-R | 5’>AACCTGATGACTCAATAGTCGAG<3’ |
|  |  | 2622-D1uF | 5’>ACGAGGGCAGCATCAACAGT<3’ |
|  |  | 2622-D1uR | 5’>TTCACGGTATGTCTTCGGTTTGTGGTGATTCCGTGTTGTG<3’ |
|  |  | 2622-D1dF | 5’>CACAACACGGAATCACCACAAACCGAAGACATACCGTGAA<3’ |
|  |  | 2622-D1dR | 5’>GGTGCTTGAGTGCTGCCATA<3’ |
|  |  | 2622-QC1-F | 5’>CATCAGGCCGAGATGTTGTATTAGCA<3’ |
|  |  | 2622-QC1-R | 5’>CTGACAATTCCCACAAACGAAGACC<3’ |
| *IDP1* | Target deletion by CRISPR/Cas9 | 2926-PAM1-F | 5’>ATCCGATTGCATCCATCTTTGCT<3’ |
|  |  | 2926-PAM1-R | 5’>AACAGCAAAGATGGATGCAATCG<3’ |
|  |  | 2926-D1uF | 5’>CGGCGTAGGTGTCAAGTGC<3’ |
|  |  | 2926-D1uR | 5’>GTCCTTGGTCATAATACCGTGAAACCACCCTTGGACTT<3’ |
|  |  | 2926-D1dF | 5’>AAGTCCAAGGGTGGTTTCACGGTATTATGACCAAGGAC<3’ |
|  |  | 2926-D1dR | 5’>TGGCTGCTAATAATAATGAC<3’ |
|  |  | 2926-QC1-F | 5’>CAACCGTGATGCCACAGATGACAAAG<3’ |
|  |  | 2926-QC1-R | 5’>AATACCCTTTGGCAAGCGTAATAGCA<3’ |
| *MTC6* | Target deletion by CRISPR/Cas9 | 4247-PAM1-F | 5’>ATCCCCATGTTTGATTCAAAGCA<3’ |
|  |  | 4247-PAM1-R | 5’>AACTGCTTTGAATCAAACATGGG<3’ |
|  |  | 4247-D1uF | 5’>TAAGGCATTGACCGATAGCA<3’ |
|  |  | 4247-D1uR | 5’>TCGGTATTAGAAGAGGGTATCAACAGGATGACAACCCACT<3’ |
|  |  | 4247-D1dF | 5’>AGTGGGTTGTCATCCTGTTGATACCCTCTTCTAATACCGA<3’ |
|  |  | 4247-D1dR | 5’>ACTGTAAATGGAGGAATGGT<3’ |
|  |  | 4247-QC1-F | 5’>TTCTATTGTCTTGAATAGGGATGTG<3’ |
|  |  | 4247-QC1-R | 5’>CATCTTGGTATGTATGACCTCCTAAT<3’ |
|  | Target mutation by CRISPR/Cas9 | 4247-PAM3-F | 5’>ATCGTTGTCATCCTGTTGTACCG<3’ |
|  |  | 4247-PAM3-R | 5'>AACCGGTACAACAGGATGACAAC<3' |
|  |  | 4247-D6uR | 5’>GGGTATGAGTGTTGGTTATATATACTAAAAAAATGATCTAGCGAATGACCG<3’ |
|  |  | 4247-D6dF | 5’>CGGTCATTCGCTAGATCATTTTTTTAGTATATATAACCAACACTCATACCC<3’ |
| *TWF1* | Target deletion by CRISPR/Cas9 | 4515-PAM1-F | 5’>ATCTCAGACAACTCTAGCTCTTG<3’ |
|  |  | 4515-PAM1-R | 5’>AACCAAGAGCTAGAGTTGTCTGA<3’ |
|  |  | 4515-D1uF | 5’>GCCATCCTCAATGATAGTCCG<3’ |
|  |  | 4515-D1uR | 5’>GATGGTTTACGCAGCAAATAGGTTCACAAACACCAGCAAA<3’ |
|  |  | 4515-D1dF | 5’>TTTGCTGGTGTTTGTGAACCTATTTGCTGCGTAAACCATC<3’ |
|  |  | 4515-D1dR | 5’>CTATGAGGAGTTCGGGAGCA<3’ |
|  |  | 4515-QC1-F | 5’>CGTTATTCCATCCTTCCTGCTTGACC<3’ |
|  |  | 4515-QC1-R | 5’>TGGTAGTAACGACGACACGCCCCTCT<3’ |
| *HRD3* | Target deletion by CRISPR/Cas9 | 4558-PAM1-F | 5’>ATCCAGATTCCTGTAGATAGATC<3’ |
|  |  | 4558-PAM1-R | 5’>AACGATCTATCTACAGGAATCTG<3’ |
|  |  | 4558-D1uF | 5’>GAATAGTCTGTTTGGCTGGTA<3’ |
|  |  | 4558-D1uR | 5’>TACCGAGGGAAAGAACTCCAACGAGGCTGTAAAGGGAC<3’ |
|  |  | 4558-D1dF | 5’>GTCCCTTTACAGCCTCGTTGGAGTTCTTTCCCTCGGTA<3’ |
|  |  | 4558-D1dR | 5’>TCAGGATCTAAGACGAAGC<3’ |
|  |  | 4558-QC1-F | 5’>CGATAGATCCCTGTTGAGCTGCGAGTAG<3’ |
|  |  | 4558-QC1-R | 5’>GATGAGGCATGGAACTCTGCACAACG<3’ |
| *CMS1* | Target deletion by CRISPR/Cas9 | 4858-PAM1-F | 5’>ATCCAAGAAGAGACCTCTAGAAG<3’ |
|  |  | 4858-PAM1-R | 5’>AACCTTCTAGAGGTCTCTTCTTG<3’ |
|  |  | 4858-D1uF | 5’>AAGTACCACCGAACAGGGC<3’ |
|  |  | 4858-D1uR | 5’>CAGGGTTCTTCTCACGAATCATCATCAGATTCCACCACAT<3’ |
|  |  | 4858-D1dF | 5’>ATGTGGTGGAATCTGATGATGATTCGTGAGAAGAACCCTG<3’ |
|  |  | 4858-D1dR | 5’>ATCTTGCGGTTTACCTTTAT<3’ |
|  |  | 4858-QC1-F | 5’>GGAAGAATTCGGGTCTTGTAGGTTG<3’ |
|  |  | 4858-QC1-R | 5’>TAAAATAGTAGAATCTTCACGGACG<3’ |
| *SSK1* | Target deletion by CRISPR/Cas9 | 4860-PAM1-F | 5’>ATCAGAATAAAGCCAAGATAGCC<3’ |
|  |  | 4860-PAM1-R | 5’>AACGGCTATCTTGGCTTTATTCT<3’ |
|  |  | 4860-D1uF | 5’>TTGGCCTGAGAATGATATTATC<3’ |
|  |  | 4860-D1uR | 5’>GGAACAGCACGATAAGAATTTGATATGGATTTACAGCTACCG<3’ |
|  |  | 4860-D1dF | 5’>CGGTAGCTGTAAATCCATATCAAATTCTTATCGTGCTGTTCC<3’ |
|  |  | 4860-D1dR | 5’>AATGAGCCGTCGTCTGGTGC<3’ |
|  |  | 4860-QC1-F | 5’>GTCGAGACCGATAGGTGTTGATCCTG<3’ |
|  |  | 4860-QC1-R | 5’>GGTGCGGTTGGATGGTAATGGTAGCG<3’ |
| *ATG1* | Target deletion by CRISPR/Cas9 | ATG1-PAM2-F | 5’>ATCATCTATCAAACCGACAATAT<3’ |
|  |  | ATG1-PAM2-R | 5’>AACATATTGTCGGTTTGATAGAT<3’ |
|  |  | ATG1-D2uF | 5’>ATACATTTCATTATTCCATCTT<3’ |
|  |  | ATG1-D2uR | 5’>AAATCATAGGGAATATAGTTGGATGATATGTATAGGCGAGGTT <3’ |
|  |  | ATG1-D2dF | 5’>AACCTCGCCTATACATATCATCCAACTATATTCCCTATGATTT<3’ |
|  |  | ATG1-D2dR | 5’>GAGTAAGTAGTAGTATTAGTCT<3’ |
|  |  | ATG1-QC2-F | 5’>TGCACGGACATTCTTGCATTC<3’ |
|  |  | ATG1-QC2-R | 5’>AGACACCCAACCTTTCTCTACG<3’ |
| *ATG8* | Directed labeling of EGFP-tag by CRISPR/Cas9 | ATG8-PAM2-F | 5’>ATCTGAGAAGAGAAAAGCGGAAT<3’ |
|  |  | ATG8-PAM2-R | 5’>AACATTCCGCTTTTCTCTTCTCA<3’ |
|  |  | EGFP/ATG8-D3F1 | 5’>TTTCTAATCTGAAGCGCATGTACTG<3’ |
|  |  | EGFP/ATG8-D1R1 | 5’>GTTCTTCTCCTTTACTCATTATCAAATAATATTATAAC<3’ |
|  |  | EGFP/ATG8-D1F2 | 5’>GTTATAATATTATTTGATAATGAGTAAAGGAGAAGAAC<3’ |
|  |  | EGFP/ATG8-D2R2 | 5’>TCTCTTCTCAAAAGGAAACTCAGACTTAAAAGCACTTTTTTTGTATAGTTCATCCATGCC  <3’ |
|  |  | EGFP/ATG8-D2F3 | 5’>AGTCTGAGTTTCCTTTTGAGAAGAGAAAAGCGGAGTCAGAAAGGATAGTACAAAAGTTCC<3’ |
|  |  | EGFP/ATG8-D1R4 | 5’>CTTCCCTCAAGTTCGTTCACAG<3’ |
|  |  | ATG8-QC1-F | 5’>ACAGCGAGCGAGTGAGCATTTG<3’ |
|  |  | ATG8-QC1-R | 5’>CAAAGTTTTGTGGGAGTCCAGC<3’ |

**Table S3 Information of the specific SNP or indel in the genome of T1 mutant**

|  | **Contigs** | **Mutation Sites** | **ORFs** | **Variance of codons** | **Variance of amino acids** |
| --- | --- | --- | --- | --- | --- |
| **CDS** | 1 | 163157 | *BEM1* | GAC->GTC | Asp > Val |
|  | 1 | 700435 | *SLH1* | GAA->AAA | Glu > Lys |
|  | 1 | 704513 | *HER2* | CAG->CTG | Gln > Leu |
|  | 1 | 768625 | *SPC97* | ATT->AAT | Ile > Asn |
|  | 1 | 895916 | *ERF4* | CAA->CAT | Gln > His |
|  | 1 | 1224656 | *PYC2* | CAC->CGC | His > Arg |
|  | 1 | 1515155 | *TFC4* | ATG->ATA | Met > Ile |
|  | 1 | 1539874 | *TOF1* | GAA->TAA | Glu > *STOP* |
|  | 2 | 85987 | *VPS10* | AAA->ACA | Lys > Thr |
|  | 2 | 159940 | *FES1* | ACG->ACT | *Synonymous* |
|  | 2 | 353985 | *SIR4* | AGA->AAA | Arg > Lys |
|  | 2 | 1270758 | *FIG4* | ATC->ATT | *Synonymous* |
|  | 2 | 844539 | *USE1* | GCC->GCA | *Synonymous* |
|  | 2 | 455693 | *ESBP6* | CTG->CTA | *Synonymous* |
|  | 3 | 699660 | *CDA2* | TGT->AGT | Cys > Ser |
|  | 3 | 859342 | *RTS3* | AAC->AAA | Asn > Lys |
|  | 3 | 1064780 | *YNR021W* | +C | *Frameshift* |
|  | 3 | 1272313 | *PDE2* | TTC->TCC | Phe > Ser |
|  | 4 | 389170 | *SEA3* | CAT->CTT | His > Leu |
|  | 4 | 409746 | *MDN1* | TTA->TCA | Leu > Ser |
|  | 4 | 536297 | *RPB5* | CAT->CCT | His > Pro |
|  | 4 | 578795 | *RPF1* | AAT->AAA | Asn > Lys |
|  | 4 | 582541 | *PDR5* | TCT->GCT | Ser > Ala |
|  | 4 | 582644 | *PDR5* | TAC->TAT | *Synonymous* |
|  | 4 | 582655 | *PDR5* | GTG->TTG | Val > Leu |
|  | 4 | 883547 | *VSH2* | AAT->AAA | Asn > Lys |
|  | 4 | 914268 | *KRI1* | TTC->TCC | Phe > Ser |
|  | 4 | 1065834 | *IDP1* | GAA->AAA | Glu > Lys |
|  | 4 | 1167172 | *DUS1* | TTA->ATA | Leu > Ile |
|  | 5 | 244881 | *DRS2* | GTT->ATT | Val > Ile |
|  | 5 | 244882 | *DRS2* | GTT->GTC | *Synonymous* |
|  | 6 | 1063497 | *MTC6* | -G | *Frameshift* |
|  | 7 | 260046 | *APL4* | GCC->GCA | *Synonymous* |
|  | 7 | 405705 | *TWF1* | ATT->GTT | Ile > Val |
|  | 7 | 510234 | *HRD3* | GCC->ACC | Ala > Thr |
|  | 7 | 876065 | *ICL2* | TGG->TTG | Trp > Leu |
|  | 8 | 192985 | *CMS1* | GAT->GCT | Asp > Ala |
|  | 8 | 197329 | *SSK1* | GAT->GCT | Asp > Ala |
|  | 8 | 206743 | *YOR022C* | ACC->ACA | *Synonymous* |
|  | 8 | 566726 | *TRM11* | AAG->AGG | Lys > Arg |
| **NCDS** | 1 | 6637 | — | C->T | — |
|  | 1 | 6645 | — | C->T | — |
|  | 1 | 6662 | — | G->A | — |
|  | 1 | 1512673 | — | -T | — |
|  | 2 | 228565 | — | G->T | — |
|  | 2 | 617051 | — | T->A | — |
|  | 2 | 726038 | — | +G | — |
|  | 2 | 1622983 | — | +G | — |
|  | 2 | 1679046 | — | G->A | — |
|  | 3 | 45047 | — | +A | — |
|  | 3 | 695918 | — | T->C | — |
|  | 3 | 860112 | — | +T | — |
|  | 3 | 897928 | — | T->G | — |
|  | 3 | 1156428 | — | +A | — |
|  | 3 | 1195762 | — | -AGGGGGGGGGGG | indel |
|  | 3 | 1319663 | — | -ACCCCCCCCCCC | indel |
|  | 4 | 609297 | — | A->G | — |
|  | 5 | 777330 | — | A->G | — |
|  | 5 | 850356 | — | C->T | — |
|  | 5 | 1048778 | — | +C | — |
|  | 6 | 704715 | — | G->T | — |
|  | 6 | 704718 | — | G->T | — |
|  | 6 | 893908 | — | +A | — |
|  | 6 | 904789 | — | +CAGCCATCA | indel |
|  | 6 | 1208884 | — | T->C | — |
|  | 6 | 1208887 | — | G->C | — |
|  | 6 | 1208898 | — | A->G | — |
|  | 7 | 268921 | — | T->G | — |
|  | 7 | 560710 | — | -AAAAAAAAAAA | indel |
|  | 8 | 163696 | — | -AAAAAAAAAAAAAT | indel |
|  | 8 | 703216 | — | A->G | — |
|  | 8 | 888049 | — | G->T | — |

Note: CDS was abbreviated from the coding DNA sequences, and NCDS was abbreviated from the non-coding DNA sequences; indel was short for insertion or deletion of oligonucleotide fragments.
